# Mechanoluminescent Materials Enable Mechanochemically Controlled Atom Transfer Radical Polymerization and Polymer Mechanotransduction

**DOI:** 10.34133/research.0243

**Published:** 2023-10-03

**Authors:** Zexuan Li, Zhenhua Wang, Chen Wang, Wenxi Li, Wenru Fan, Ruoqing Zhao, Haoyang Feng, Dengfeng Peng, Wei Huang

**Affiliations:** ^1^ Frontiers Science Center for Flexible Electronics, Institute of Flexible Electronics, Northwestern Polytechnical University, Xi’an 710072, China.; ^2^ School of Materials Science and Engineering, Northwestern Polytechnical University, Xi’an 710072, China.; ^3^ Key Laboratory of Optoelectronic Devices and Systems of Ministry of Education and Guangdong Province, College of Physics and Optoelectronic Engineering, Shenzhen University, Shenzhen 518060, China.

## Abstract

Organic mechanophores have been widely adopted for polymer mechanotransduction. However, most examples of polymer mechanotransduction inevitably experience macromolecular chain rupture, and few of them mimic mussel’s mechanochemical regeneration, a mechanically mediated process from functional units to functional materials in a controlled manner. In this paper, inorganic mechanoluminescent (ML) materials composed of CaZnOS-ZnS-SrZnOS: Mn^2+^ were used as a mechanotransducer since it features both piezoelectricity and mechanolunimescence. The utilization of ML materials in polymerization enables both mechanochemically controlled radical polymerization and the synthesis of ML polymer composites. This procedure features a mechanochemically controlled manner for the design and synthesis of diverse mechanoresponsive polymer composites.

## Introduction

Polymer mechanotransduction has emerged to convert the input mechanical energy into productive chemical or light signals among synthetic polymers [1–6]. Normally, force-responsive groups (mechanophores) are inserted into polymer chains to undergo a mechanochemical reaction when exposed to external force [7–14], giving rise to mechanochromism, mechanoluminescence, mechanocatalysis, etc [15–25]. Although elegant designs have been increasingly developed in the past decade, most examples of polymers containing mechanophore inevitably experience bond rupture [26–32], and few of them mimic mussel’s mechanochemical regeneration, a mechanically mediated process from monomer to biopolymer in a controlled manner [33–34].

Recently, as an alternative mechanotransducer to mechanophore in synthetic polymers, piezoelectric materials have gained increasingly interest for mechanically controlled radical polymerizations including atom transfer radical polymerization (ATRP) and reversible addition fragmentation chain transfer polymerization [35–54]. These methodologies have been demonstrated to synthesize well-defined polymers with predicted molecular weight, high chain-end fidelity, and narrow molecular distribution [55]. Taking advantage of this chemistry, Esser-Kahn and colleagues [56–58] developed a series of mechanochemically strengthened composites comprising synthetic thiol-based polymers and piezoelectric nanoparticles, which would be cross-linked to ultrasonic agitation or mechanical vibration. Although these mechanochemically formed polymer networks could be recovered into linear polymer chains under reduction, they can hardly be used for the repeatable mechanoresponsive process because thiols are not stable and highly reactive to oxidation [59–61].

**Fig. 1.**
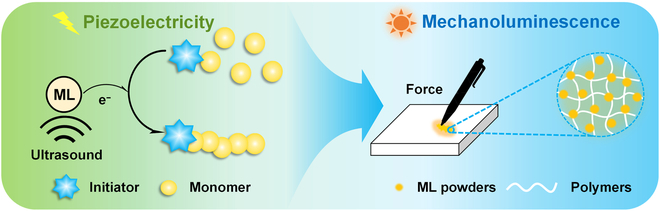
Dual roles of ML materials.

Semiconductor heterojunction, combined with its piezoelectric and semiconductive properties, has done a lot of original work in moderating elastic mechanoluminescence [62–66]. In this study, ternary-heterojunction ML CaZnOS-ZnS-SrZnOS: Mn^2+^ was designed as a mechanotransducer to conduct mechanically controlled ATRP (mechano-ATRP) for the synthesis of repeatably responsive mechanoluminescent (ML) composites. ML materials were synthesized by high-temperature solid-phase reactions, and their luminescence properties were reported in previous work [65]. ML materials were used in this work because it has 2 functions: piezoelectricity and mechanoluminescence. The utilization of piezoelectricity facilitates electron transfer under mechanical forces to convert the deactivators (Cu^II^/L) into the activators (Cu^I^/L), thus activating polymerization to synthesize desired polymers in a controlled manner (Fig. 1). In addition, the resultant polymer composites containing ML powders possess the ability to emit orange luminescence upon force. This procedure enables the synthesis of various ML composites with well-defined polymers, tunable compositions, and mechanical performance.

## Results

In a preliminary experiment, mechano-ATRP of methyl acrylate (MA) was attempted by using ML materials as the mechanotransducer for the mechanoelectrical reduction of Cu (II) to Cu (I). A Schlenk flask was charged with 200/1/0.03/0.12 molar ratio of MA, ethyl α-bromoisobutyrate (EBiB) (initiator), CuBr_2_/TPMA (catalyst), dimethyl sulfoxide (DMSO), and ML materials under nitrogen (N_2_). The mixture was then subjected to ultrasonic irradiation at 45 kHz. As shown in (Table), the conversion of polymerization approached 92% after 6-h ultrasonic agitation under identical conditions, yielding polymethyl acrylate (PMA) with *M*_n_ = 10,100 and *Đ* = 1.07 (entry 2). While the ultrasound (US) was replaced by ultraviolet (UV) irradiation, the polymerization could not proceed (entry 1), indicating the advantage of the US over UV light toward the control of the heterogeneous polymerization process.

**Table.** Mechano-ATRP for the synthesis of homopoly(meth)acrylates with different DP_T_

Entry ^a^	Monomer	*D*P_T_	*T* (h)	ML (wt%)	Conversion ^b^	*M*_n,th_^c^	*M*_n, GPC_^d^	*Đ* ^d^
1 ^e^	MA	200	6	4.8	7%	1,400	600	1.24
2	MA	200	6	4.8	92%	16,000	10,100	1.07
3	MA	400	6	4.8	87%	30,100	24,900	1.09
4	MA	800	6	4.8	78%	53,800	39,300	1.21
5	MMA	200	10	4.8	61%	12,400	14,200	1.23
6	MMA	400	10	4.8	48%	19,300	14,700	1.26
7	MMA	800	10	4.8	37%	29,800	28,200	1.31
8 ^f^	BA	200	4	4.8	68%	17,800	14,800	1.15
9 ^f^	MEMA	200	4	4.8	50%	14,600	15,400	1.19
10	EA	200	4	4.8	64%	13,000	12,400	1.10
11 ^g^	BMA	200	6	4.8	38%	10,900	15,400	1.33

^a^ Reaction conditions: [Monomer]_0_/[CuBr_2_]_0_/[TPMA]_0_/[EBiB]_0_ = *X*/0.03/0.12/1 (*X* = 200, 400, or 800), 4.8 wt% ML powders, 50% (v/v) DMSO, 25 °C, 45 kHz, 300 W.

^b^ Conversion was determined using ^1^H NMR.

^c^ Calculated based on conversion (i.e., *M*_n,th_ = *M*_EBiB_ + [Monomer]_0_/[EBiB]_0_ × conversion × *M*_monomer_).

^d^ Determined by GPC in THF, based on linear PMMA as the calibration standard.

^e^ The polymerization was conducted in UV light (365 nm).

^f^ The polymerization was conducted in DMF.

^g^ The polymerization was conducted in mixed solvents (DMF/anisole = 1/1).

**Fig. 2.**
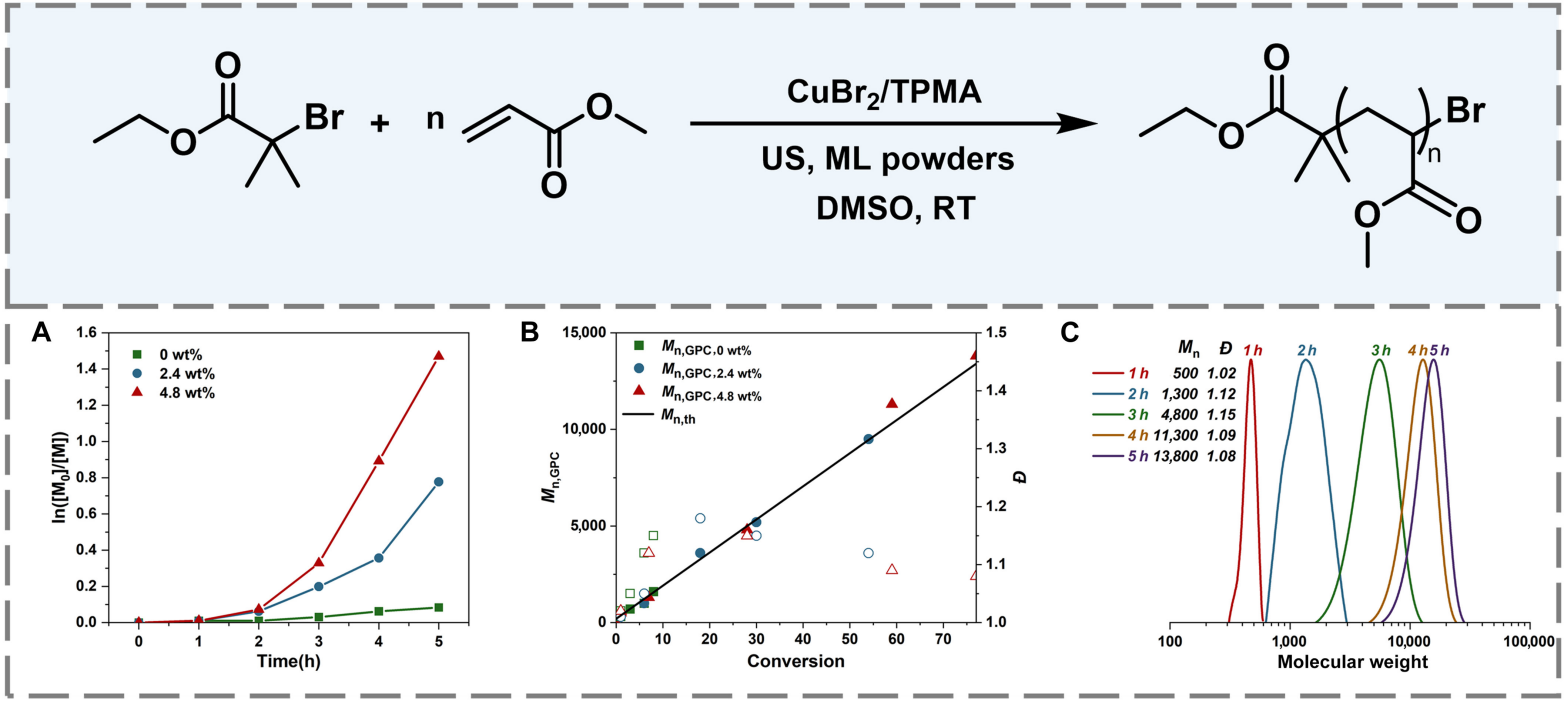
(A) Kinetics of mechano-ATRP with different ML materials (red: 0 wt%; green: 2.4 wt%; blue: 4.8 wt%). (B) The number-average molecular weight and dispersity Đ. (C) GPC traces of polymers. Reaction conditions: [MA]_0_/[CuBr_2_]_0_/[TPMA]_0_/[EBiB]_0_ = 200/0.03/0.12/1, 50% (v/v) DMSO, 25 °C, 45 kHz, 300 W.

To further validate the role of ML materials in polymerization, the effect of ML loadings on mechano-ATRP was investigated. As shown in Fig. 2, all the reactions revealed an induction period, indicating a slow activation in the beginning. This is different from the previous piezoelectric systems (ZnO, BaTiO_3_), potentially because of the lower surface area for the microscale ML materials compared to the nanoscale piezoelectric particles. The polymerization reached only 9% conversion in the absence of ML materials after 5 h of ultrasonic irradiation (Fig. S2). When the loading of ML materials increased to 2.4 wt%, the polymerization gave 54% conversion after 5 h of ultrasonic irradiation, giving a PMA with *M*_n_ = 9,500 and *Đ* = 1.12 (Figs. S3 and S5). The conversion increased to 77% with 4.8-wt% loading of ML materials, affording a polymer with *M*_n_ = 13,800 and *Đ* = 1.08 (Fig. S4). These results showed that ML materials are essential to the mechanically controlled activation and elevation of polymerization.

To examine whether this procedure can be used to synthesize polymers with predicted molecular weight, mechano-ATRP of MA targeting various degrees of polymerization (*DP*_T_) was conducted. EBiB and CuBr_2_/TPMA changed depending on the target *DP*_T_. As shown in Table, when *DP*_T_ was set at 200, the conversion of MA reached 92%, giving a PMA with *M*_n_ = 10,100 and *Đ* = 1.07 after 6 h of US irradiation (entry 2).

Mechano-ATRP with *DP*_T_ = 400 attained 87% conversion, giving a PMA with *M*_n_ = 24,900 and *Đ* = 1.09 (entry 3). The polymerization reaction targeting *DP*_T_ = 800 achieved 78% conversion, affording PMA with *M*_n_ = 39,300 and *Đ* = 1.12 (entry 4). Mechano-ATRP of methyl methacrylate (MMA) with *DP*_T_ = 200 gave 64% conversion, producing polymethyl methacrylate (PMMA) homopolymer with *M*_n_ = 16,300 and *Đ* = 1.29 after 10 h of ultrasonic irradiation (entry 5). The conversion of polymerization targeting *DP*_T_ = 400 reached 56%, giving well-defined PMMA with *M*_n_ = 18,600 and *Đ* = 1.29 (entry 6). When *DP*_T_ was set at 800, the conversion approached 37%, yielding PMMA with *M*_n_ = 28,200 and *Đ* = 1.31 (entry 7).

Furthermore, this procedure was expanded into the polymerization of other monomers including butyl acrylate (BA), butyl methacrylate (BMA), 2-methoxyethyl methacrylate (MEMA), and ethyl acrylate (EA) in Table. All monomers were successfully polymerized to give well-defined polymers under ultrasonic irradiation. The experimental molecular weight of all the synthesized polymers matched well with the theoretical value accompanied with narrow molecular weight distribution.

To confirm the retention of chain-end functionality in the mechano-ATRP procedure, chain extension reaction was carried out. The first block was conducted under the standard conditions, yielding a PMA-Br macroinitiator with *M*_n_ = 4,000 and *Đ* = 1.15 at 23% conversion. The chain extension of PMA-Br with EA was further conducted. After 4 h of ultrasonic bath, the chain-extended polymerization of PMA-*b*-PEA-Br was prepared with *M*_n_ = 8,400 and *Đ* = 1.05 as shown in Fig. S20. To further examine the chain-end retention, matrix-assisted laser desorption/ionization-time of flight (MALDI-TOF) mass spectrometry was used. The spectrum showed a steady increase in molecular mass as shown in Fig. S21, which is consistent with the molecular weight of MA unit (*M*_n_ = 86). The strongest peak revealed a molecular weight of 8,271 which matched well with the corresponding gel permeation chromatography (GPC) trace (*M*_n_ = 8,700). Another distribution at 8,255 can be attributed to the removal of a methyl group by laser irradiation [67–68]. In conclusion, the MALDI-TOF spectra confirmed the high chain-end fidelity of PMA synthesized via this mechano-ATRP procedure.

**Fig. 3.**
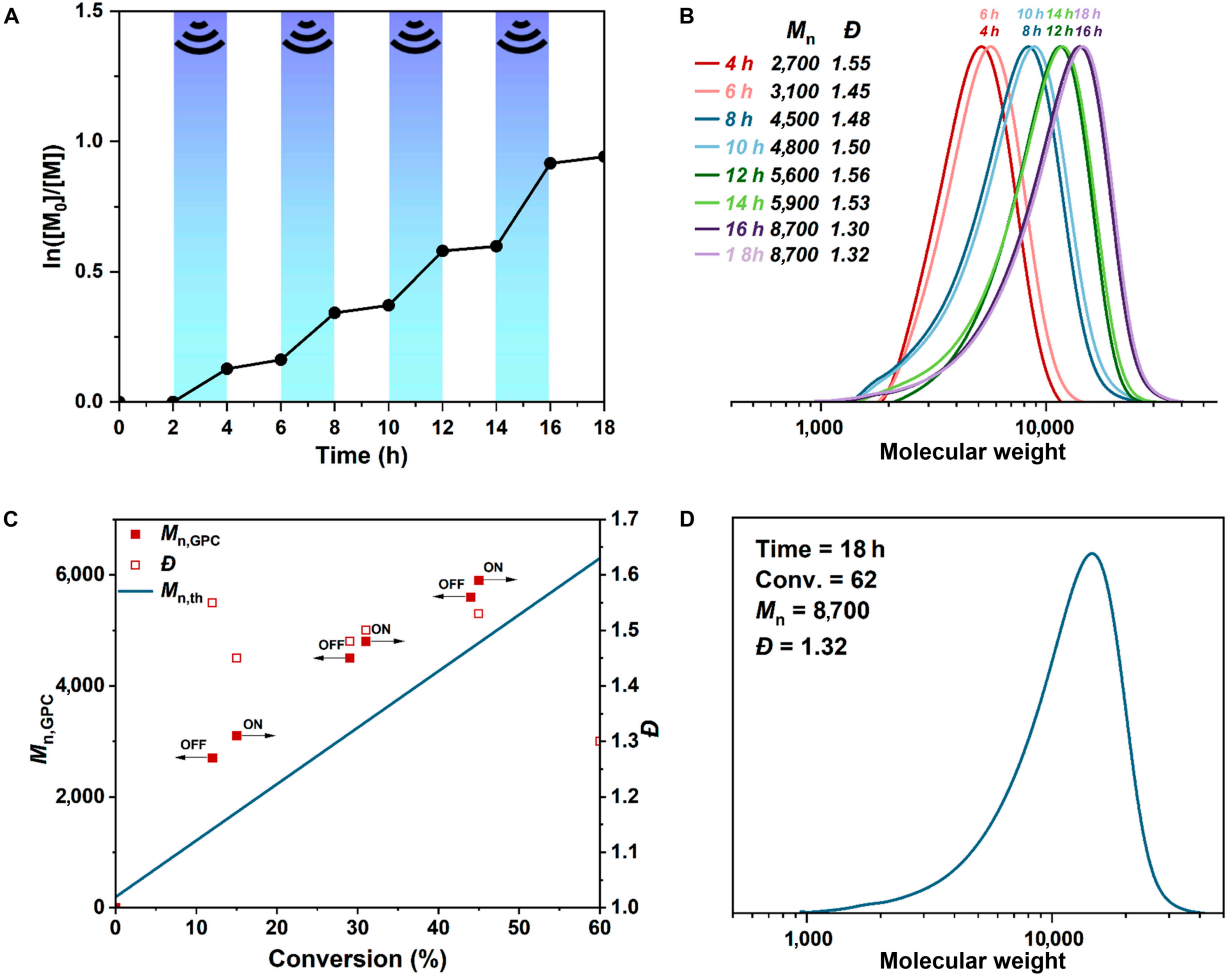
(A) Temporal control in mechano-ATRP of MMA by switching US between the on/off states in the presence of the 4.8-wt% ML powders. (B) GPC analysis of the resulting polymer during 18 h (4^1^/_2_ cycles of light on/off periods). (C) Molecular weight and conversion rate, target molecular weight. (D) GPC traces of the resulting polymer after 18 h. Reaction conditions: [MMA]_0_/[CuBr_2_]_0_/[TPMA]_0_/[EBiB]_0_ = 100/0.03/0.12/1, 50% (v/v) DMSO, 25 °C, 45 kHz, 300 W.

To examine the temporal control over polymerization, ultrasonic agitation was switched on/off for several cycles. Samples were periodically withdrawn from the vessel and analyzed by GPC to monitor the evolution of polymer *M*_n_ over time and analyzed by ^1^H nuclear magnetic resonance (NMR) spectroscopy to calculate monomer conversion (Fig. S22). As shown in Fig. 3, the growth of polymer molecular chains can be well controlled by ultrasonic irradiation. The molecular weight of the polymer produced a remarkable increase under US, whereas during the US-off periods negligible monomer conversion was observed (Fig. 3A and B). Excellent control over mechano-ATRP of MMA throughout this US on/off cycle, and the experimental molecular weight of the polymer is slightly higher than the theoretical value (*M*_n_ = 8,700, *M*_n, th_ = 6,300), potentially because of the low initiation efficiency of EBiB for MMA (Fig. 3C and D).

**Fig. 4.**
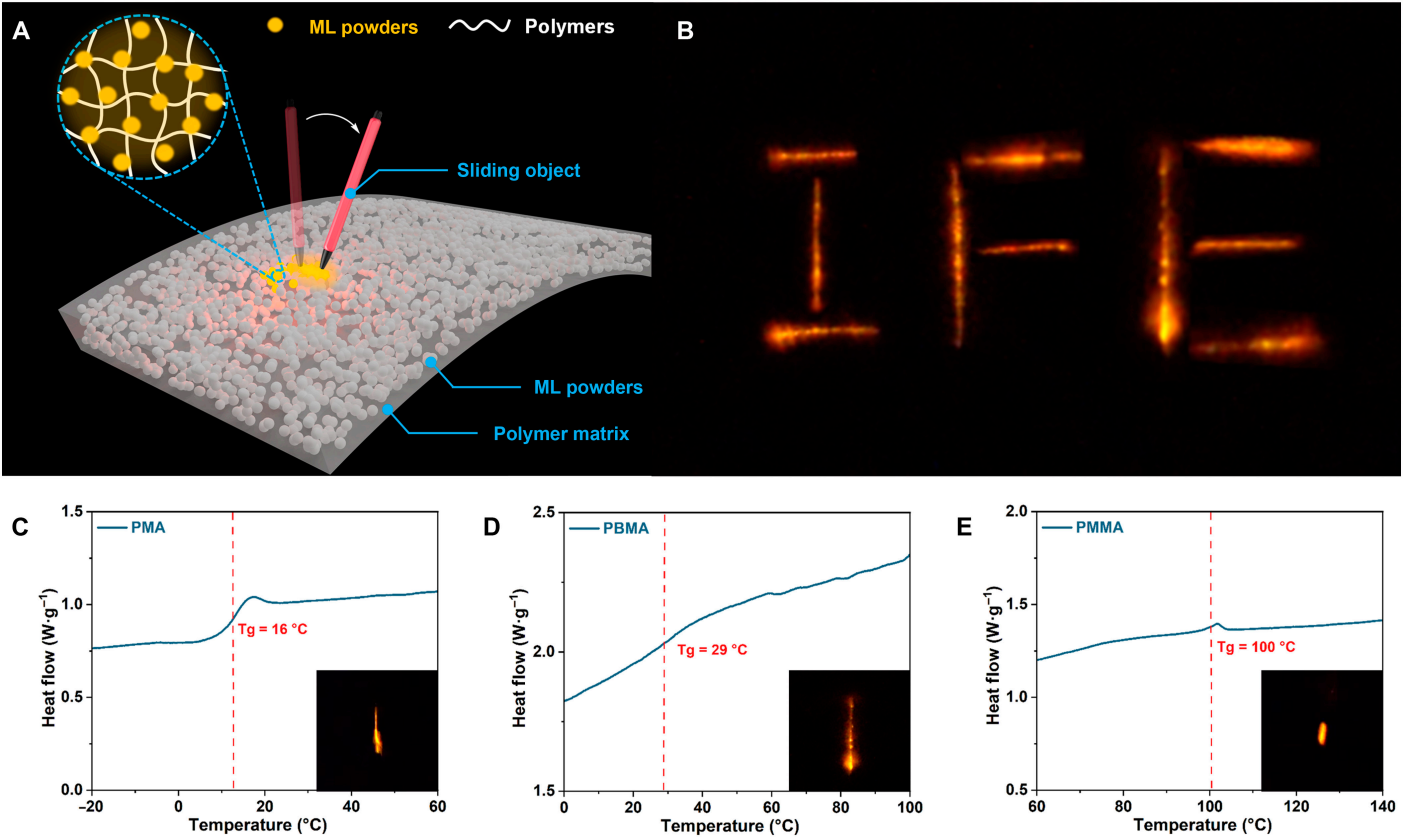
(A) Structure of the ML polymer composites. (B) Superimposed image showing the complete trajectory of letters “IFE”. (C to E) DSC trace of PMA-, PBMA-, and PMMA-based composites and images of mechanoluminescence triggered by scraping with a pen.

To study the ML properties, a series of polymer composites with different glass transition temperatures (*T*_g_) were synthesized via the standard mechano-ATRP procedure (Fig. 4C to E). *T*_g_ of the ML composites increased from 16 to 100 °C when the monomer changed from MA to MMA. All the composites emitted yellow light upon force repeatably. The emission peak of the ML spectrum was located at 600 nm (Fig. S26). A camcorder was employed to capture the luminescence induced by the sliding object and intercept photographs of the ML materials producing the mechanoluminescence trajectory. When combined and superimposed together, the photos yielded an overall trajectory of 3 complete letters “IFE” in Fig. 4B. In addition, the thermal properties of the ML polymer composites were further investigated by thermogravimetric analysis (Fig. S28). The decomposition temperature (*T*_d_) corresponding to 5% weight loss of the composites varied from 270 °C (PMMA) to 360 °C (PMA). The weight fraction of inorganic ML powders approached 28.7 wt% for PMA-based composites, while in the case of PMMA- and PBMA-based composites, the amount of inorganic ML powders was 12.1 and 13.4 wt% (Table S1).

## Discussion

In summary, the intriguingly piezoelectric and ML CaZnOS-ZnS-SrZnOS: Mn^2+^ has been demonstrated to facilitate the mechanoactivation of the ATRP process and construct mechanoresponsive polymer composites. This mechano-ATRP procedure with ML materials is compatible with a broad scope of monomers, providing well-defined polymers with high chain-end fidelity and predetermined molecular weights. In addition, the polymerization process can be temporally controlled by switching the ultrasonic bath on and off. Taking advantage of this mechano-ATRP procedure, repeatably ML polymer composites with various *T*_g_ have been successfully prepared. This work provides new sight into the design of ML polymers and lay a foundation for the development of mechano-photoresponsive polymers in the future.

## Materials and Methods

### Materials

ML powders, TPMA was synthesized according to previous work. Others of the chemicals were purchased from commercial sources. EBiB (99%), copper bromide (CuBr_2_, 99%), DMSO (>99%), N, N-dimethylformamide (DMF, >99%), anisole (>99%), tetrahydrofuran (THF, ≥99.9%), 2,5-dihydroxybenzoic acid (2,5-DHB, 98%) and deuterated chloroform (CDCl_3_, 99%) were purchased from Rhawn. MA (99%), MMA (99%), BA (98%), BMA (99%), MEMA (98%), and EA (99%) were purchased from Adamas and purified by passing through a column of basic alumina to remove inhibitors. All chemicals were used as received unless otherwise indicated.

### Characterization

All the monomer conversions were performed via ^1^H NMR in CDCl_3_ using a Bruker Avance Neo 500 MHz spectrometer at 25 °C. The molecular weights and dispersity were determined using an Agilent 1260 high performance liquid chromatography system equipped with a G7110B pump and a G7162A refractive index detector. All polymers were performed in THF solution (>99.8%, high performance liquid chromatography) at 35 °C with an elution rate of 0.5 ml min^−1^. The apparent molecular weights were determined on single photoluminescence gel MIXED-C columns using linear poly (methyl methacrylate) standards. Prior to the analysis, the sample was diluted with THF and filtered through a column of neutral alumina to remove Cu (II) and then filtered through a 0.22-μm nylon (NY) membrane filter before injecting it into the GPC columns (injection volume: 10 μl). Mechano-induced polymerization was performed in an ultrasonic bath (KUNSHAN KQ-300VDE, 45 kHz, 300 W), and the temperature was maintained in the range of 20 to 30 °C by immersing a hollow Cu cooling coil with circulating running water in the bath. The reaction device was placed in the ultrasonic bath for 1 h, and the temperature was observed until the temperature stabilized. All the conversions were measured using ^1^H NMR and the molecular weights were determined via GPC. The dried samples were heat pressed using an HP-100 heat-pressing machine (Hefei Kejing Material Technology Co., Ltd.) at 120 °C and 10 MPa for 10 min. The samples cooled to 25 °C spontaneously to give a hybrid material with 2-mm thickness. The ML spectra were collected from a spectrometer of Ocean Optic QE6500 with a liquid-nitrogen-cooled power detector (S7031-1006, HANANATSU). The scanning electron microscope images were obtained by a Zeiss Gemini SEM 300. The x-ray-induced luminescence patterns were obtained using an Omni-λ 300i spectrograph (Zolix) equipped with an x-ray tube (Model RACA-3, Zolix Instruments Co. Ltd., Beijing, China). The photoluminescence images were obtained using a Hitachi F-4600 spectrophotometer equipped with an R928 photomultiplier detector. MALDI-TOF mass spectrometer was from Bruke, Germany. The MALDI instrument was equipped with a 337-nm pulsed nitrogen laser (laser intensity of 50 Hz). The number of laser irradiations was 100 for all mass spectra (delay time of 190 ns), with a 20-kV acceleration voltage. MALDI experiment was carried out using 2,5-DHB as the matrix. The matrix solution was prepared by dissolving 40 mg of 2,5-DHB in 1 ml of THF. Thermogravimetric analysis measurements were performed using a TG 209 F3 (German NETZSCH) and 80-μl aluminum oxide pans. The measurements were done under nitrogen and/or oxygen (quality 5.0) atmosphere and a gas flow of 50 ml/min for both gases. The heating rates was 10 °C/min.

### General procedure for ultrasonication-mediated ATRP of MA

One millilter of MA (0.96 g, 11 mmol, 200 equiv.), 8.2 μl of EBiB (10.8 mg, 0.056 mmol, 1 equiv.), 0.72 mg of CuBr_2_ (3.2 μmol, 0.03 equiv.), 3.7 mg of TPMA (12.9 μmol, 0.12 equiv.), 100-mg ML, and 1 ml of DMSO were added to a 10-ml Schlenk flask. The oxygen inside the flask was removed by N_2_ bubbling method for 15 min and sealed. The reaction was placed in US bath for polymerization. The reaction was removed from the ultrasonic bath and exposed to air to quench the reaction.

After the reaction, samples were withdrawn from the bottle to analyze the conversion by ^1^H NMR and number-average molecular weight (*M*_n_) and dispersity (*Đ*) by GPC. THF (4 ml) was added to dilute a small part extracted from reaction mixture that then passed through neutral Al_2_O_3_ to remove catalysts. Then, 1.5 ml of sample was removed to test GPC.

### Synthesis of a PMA-Br macroinitiator

Five milliliters of MA (4.8 g, 55 mmol, 200 equiv.), 41 μl of EBiB (54 mg, 0.28 mmol, 1 equiv.), 3.6 mg of CuBr_2_ (8.5 μmol, 0.03 equiv.), 10.0 mg of TPMA (34 μmol, 0.12 equiv.) 500-mg ML, and 5 ml of DMSO were added to a 10-ml Schlenk flask. The reaction flask was sealed and degassed by N_2_ purging for 15 min. The reaction was placed in US bath for polymerization. After 2 h, conversion, number-average molecular weight (*M*_n_), and dispersity (*Đ*) were tested in the same way as described above. The macroinitiator was obtained by precipitation in mixture of cooled MeOH/H_2_O (6/1, v/v) and dried under vacuum at 60 °C. Synthesized PMA-Br homopolymer could be used for subsequent chain extension reaction.

### Chain extension reaction of PMA-Br with EA

One milliliter of EA (0.92 g, 9.2 mmol, 200 equiv.), 184 mg of the macroinitiator (PMA-Br, 46 μmol, 1 equiv.), 0.31 mg of CuBr2 (1.4 μmol, 0.03 equiv.), 1.6 mg of TPMA (5.5 μmol, 0.12 equiv.) 100-mg ML and 1 ml of DMSO were added to a 10-ml Schlenk flask. The oxygen inside the flask was removed by N_2_ bubbling method for 15 min and sealed. The reaction was exposed to ultrasonic agitation for 4 h. Samples were withdrawn from the vial to analyze the conversion using ^1^H NMR and the number-average molecular weight (*M*_n_) and dispersity (*Đ*) using GPC with THF as an eluent using a linear PMMA standard.

### Synthesis of ML hybrid material with BMA

Four milliliters of BMA (3.6 g, 25.2 mmol, 400 equiv.), 9.2 μl of EBiB (12.3 mg, 63.1 μmol, 1 equiv.), 0.42 mg of CuBr_2_ (1.8 μmol, 0.03 equiv.), 2.18 mg of TPMA (7.5 μmol, 0.12 equiv.), 400-mg ML, and 4 ml of mixed solvents (DMF/anisole = 1/1) were added to a 25-ml Schlenk flask. The reaction flask was sealed and degassed by N_2_ purging for 30 min. Reactions were placed in an US bath for 8 h. Then, conversion, number-average molecular weight (*M*_n_), and dispersity (*Đ*) were tested in the same way as described above. The polymer was obtained by precipitation in mixture of cooled MeOH/H_2_O (4/1, v/v) and dried under vacuum at 60 °C. The hybrid material was prepared by compression molding at 120 °C.

### Synthesis of ML hybrid material with MA

Four milliliters of MA (3.8 g, 44.5 mmol, 400 equiv.), 16.3 μl of EBiB (21.7 mg, 111.2 μmol, 1 equiv.), 0.74 mg of CuBr_2_ (3.3 μmol, 0.03 equiv.), 3.9 mg of TPMA (13.3 μmol, 0.12 equiv.), 400-mg ML, and 4 ml of DMSO were added to a 25-ml Schlenk flask. The reaction flask was sealed and degassed by N_2_ purging for 30 min. Reactions were placed in an US bath for 4 h. Then, conversion, number-average molecular weight (*M*_n_), and dispersity (*Đ*) were tested in the same way as described above. The polymer was obtained by precipitation in mixture of cooled MeOH/H_2_O (3/1, v/v) and dried under vacuum at 60 °C. The hybrid material was prepared by compression molding at 130 °C.

### Synthesis of ML hybrid material with MMA

Four milliliters of methyl methacrylate (MMA, 3.8 g, 37.7 mmol, 400 equiv.), 13.8 μl of EBiB (18.3 mg, 94.1 μmol, 1 equiv.), 0.63 mg of CuBr_2_ (2.8 μmol, 0.03 equiv.), 3.3 mg of TPMA (11.3 μmol, 0.12 equiv.), 400-mg ML, and 4 ml of DMSO were added to a 25-ml Schlenk flask. The reaction flask was sealed and degassed by N_2_ purging for 30 min. Reactions were placed in an US bath for 14 h. Then, conversion, number-average molecular weight (*M*_n_), and dispersity (*Đ*) were tested in the same way as described above. The polymer was obtained by precipitation in a mixture of cooled MeOH/H_2_O (2/1, v/v) and dried under vacuum at 60 °C. The hybrid material was prepared by compression molding at 150 °C.

## Data Availability

All data needed to evaluate the conclusions in the paper are present in the paper or the Supplementary Materials.
