# Supplementary material for: Mechanoluminescent Materials Enable Mechanochemically Controlled Atom Transfer Radical Polymerization and Polymer Mechanotransduction
Source: Research (Wash D C). 2023 Oct 3;6:0243. doi: 10.34133/research.0243 (PMC10546606; doi:10.34133/research.0243)
Supplement: Supplementary 1 — Figs. S1 to S28 Table S1 [file research.0243.f1.zip › Supplementary Materials.docx]

**Supplementary Materials**

Mechanochemically Controlled Atom Transfer Radical Polymerization for Mechanoluminescent Composites

Zexuan Li^1^†, Zhenhua Wang^1^*†, Chen Wang^1^, Wenxi Li^2^, Wenru Fan^1^, Ruoqing Zhao^1^, Haoyang Feng^1^, Dengfeng Peng^3^*, Wei Hang^1^*

^1^ Frontiers Science Center for Flexible Electronics, Institute of Flexible Electronics, Northwestern Polytechnical University, Xi’an 710072, China.

*^2^* School of Materials Science and Engineering, Northwestern Polytechnical University, Xi’an 710072, China.

^3^ Key Laboratory of Optoelectronic Devices and Systems of Ministry of Education and Guangdong Province, College of Physics and Optoelectronic Engineering, Shenzhen University, Shenzhen 518060, P. R. China.

Corresponding Author

* iamwhuang@nwpu.edu.cn

* iamzhwang@nwpu.edu.cn

[* pengdengfeng@szu.edu.cn](mailto:*%20pengdengfeng@szu.edu.cn%20)

**EXPERIMENTAL SECTION**

**Materials.**

Mechanoluminescent (ML) powder^1^, Tris (2-pyridinylmethyl) amine (TPMA)^2^ was synthesized according to previous work. Others of the chemicals were purchased from commercial sources. Ethyl α-bromoisobutyrate (EBiB, 99%), copper bromide (CuBr_2_, 99%), dimethyl sulfoxide (DMSO, >99%), N, N-Dimethylformamide (DMF, >99%), anisole (>99%), tetrahydrofuran (THF, HPLC, ≥99.9%), 2,5-dihydroxybenzoic acid (2,5-DHB, 98%) and deuterated chloroform (CDCl_3_, 99%) were purchased from Rhawn. Methyl acrylate (MA, 99%), methyl methacrylate (MMA, 99%), butyl acrylate (BA, 98%), butyl methacrylate (BMA, 99%), 2-methoxyethyl methacrylate (MEMA, 98%) and ethyl acrylate (EA, 99%) were purchased from Adamas and purified by passing through a column of basic alumina to remove inhibitors. All chemicals were used as received unless otherwise indicated.

**Characterization**

All the monomer conversions were performed *via* ^1^H NMR in CDCl_3_ using a Bruker Avance Neo 500 MHz spectrometer at 25 °C. The molecular weights and dispersity were determined using an Agilent 1260 HPLC system equipped with a G7110B pump and a G7162A refractive index detector. All polymers were performed in tetrahydrofuran solution (>99.8%, HPLC) at 35 °C with an elution rate of 0.5 mL min^−1^. The apparent molecular weights were determined on single PLgel MIXED-C columns using linear poly (methyl methacrylate) standards. Prior to the analysis, the sample was diluted with THF and filtered through a column of neutral alumina to remove Cu (II) and then filtered through a 0.22 μm nylon (NY) membrane filter before injecting it into the GPC columns (injection volume: 10 μL). Mechano-induced polymerization was performed in an ultrasonic bath (KUNSHAN KQ-300VDE, 45 kHz, 300 W), and the temperature was maintained in the range of 20–30 °C by immersing a hollow Cu cooling coil with circulating running water in the bath. Place the reaction device in the ultrasonic bath for one hour to observe the temperature and wait for the temperature to stabilize. All the conversions were measured using ^1^H NMR and the molecular weights were determined via GPC. The dried samples were heat pressed using an HP-100 heat-pressing machine (Hefei Kejing Material Technology Co., Ltd.) at 120 °C and 10 MPa for 10 min. The samples cooled to 25 °C spontaneously to give a hybrid material with 2 mm thickness. The ML spectra were collected from a spectrometer of Ocean Optic QE6500 with a liquid-nitrogen-cooled power detector (S7031-1006, HANANATSU). The Scanning Electron Microscope (SEM) images were obtained by a Zeiss Gemini SEM 300. The X-ray-induced luminescence (XIL) patterns were obtained using an Omni-λ 300i spectrograph (Zolix) equipped with an X-ray tube (Model RACA-3, Zolix Instruments Co. ltd., Beijing, China). The photoluminescence (PL) images were obtained using a Hitachi F-4600 spectrophotometer equipped with an R928 photomultiplier detector. MALDI-TOF-MS from Bruke, Germany, The MALDI instrument was equipped with a 337 nm pulsed nitrogen laser (laser intensity of 50 Hz). The number of laser irradiations was 100 for all mass spectra (delay time of 190 ns), with a 20 kV acceleration voltage. MALDI experiment was carried out using 2,5-dihydroxybenzoic acid (2,5-DHB) as the matrix. The matrix solution was prepared by dissolving 40 mg of 2,5-DHB in 1 mL of THF.

**General procedure for ultrasonication-mediated ATRP of MA**

1.00 mL of methyl acrylate (MA, 0.96 g, 11 mmol, 200 equiv.), 8.2 μL of EBiB (10.8 mg, 0.056 mmol, 1 equiv.), 0.72 mg of CuBr_2_ (3.2 μmol, 0.03 equiv.), 3.7 mg of TPMA (12.9 μmol, 0.12 equiv.), 100 mg ML and 1 mL of DMSO were added to a 10 mL Schlenk flask. The oxygen inside the flask was removed by N_2_ bubbling method for 15 min and sealed. The reaction was placed in ultrasound bath for polymerization. The reaction was removed from the ultrasonic bath and exposed to air to quench the reaction.

After the reaction, samples were withdrawn from the bottle to analyze the conversion by ^1^H NMR and number-average molecular weight (*M*_n_) and dispersity (*M*_w_/*M*_n_) by GPC. 4 mL of THF was added to dilute a small part extracted from reaction mixture that then passed through neutral Al_2_O_3_ to remove catalysts. Then, remove 1.5 ml of sample to test GPC.

**Synthesis of a PMA-Br macroinitiator**

5.00 mL of methyl acrylate (MA, 4.8 g, 55 mmol, 200 equiv.), 41 μL of EBiB (54 mg, 0.28 mmol, 1 equiv.), 3.6 mg of CuBr_2_ (8.5 μmol, 0.03 equiv.), 10.0 mg of TPMA (34 μmol, 0.12 equiv.) 500 mg ML and 5 mL of DMSO were added to a 10 mL Schlenk flask. The reaction flask was sealed and degassed by N_2_ purging for 15 min. The reaction was placed in ultrasound bath for polymerization. After 4 h, conversion, number-average molecular weight (*M*_n_) and dispersity (*M*_w_/*M*_n_) were measured in the same way as described above. The macroinitiator was obtained by precipitation in mixture of cooled MeOH/H_2_O (6/1, v/ v) and dried under vacuum at 60 °C. Synthesized PMA-Br homopolymer could be used for subsequent chain extension reaction.

**Chain extension reaction of PMA-Br with EA**

1.6 mL of ethyl acrylate (EA, 1.5 g, 14.7 mmol, 200 equiv.), 727 mg of the macroinitiator (PMA-Br, 66 μmol, 1 equiv.), 0.42 mg of CuBr_2_ (1.9 μmol, 0.03 equiv.), 2.2 mg of TPMA (7.5 μmol, 0.12 equiv.) 160 mg ML and 1.6 mL of DMSO were added to a 10 mL Schlenk flask. The oxygen inside the flask was removed by N_2_ bubbling method for 15 min and sealed. The reaction was exposed to ultrasonic agitation for 7 h. Samples were withdrawn from the vial to analyze the conversion using ^1^H NMR and the number-average molecular weight *M*_n_ and dispersity (*Đ*) using GPC with THF as an eluent using a linear PMMA standard.

**Synthesis of PBA-based mechanoluminescent hybrid materials**

4 mL of butyl methacrylate (BMA, 3.6 g, 25.2 mmol, 400 equiv.), 9.2 μL of EBiB (12.3 mg, 63.1 μmol, 1 equiv.), 0.42 mg of CuBr_2_ (1.8 μmol, 0.03 equiv.), 2.18 mg of TPMA (7.5 μmol, 0.12 equiv.) 400 mg ML and 4 mL of mixed solvents (DMF/Anisole = 1/1) were added to a 25 mL Schlenk flask. The reaction flask was sealed and degassed by N_2_ purging for 30 min. Reactions were placed in an ultrasound bath for 8 hours. Then, conversion, number-average molecular weight (*M*_n_) and dispersity (*M*_w_/*M*_n_) were measured in the same way as described above. The polymer was obtained by precipitation in mixture of cooled MeOH/H_2_O (4/1, v/ v) and dried under vacuum at 60 °C. The product was prepared by compression molding at 120 °C.

**Synthesis of PMA-based mechanoluminescent hybrid materials**

4 mL of methyl acrylate (MA, 3.8 g, 44.5 mmol, 400 equiv.), 16.3 μL of EBiB (21.7 mg, 111.2 μmol, 1 equiv.), 0.74 mg of CuBr_2_ (3.3 μmol, 0.03 equiv.), 3.9 mg of TPMA (13.3 μmol, 0.12 equiv.) 400 mg ML, and 4 mL of DMSO were added to a 25 mL Schlenk flask. The reaction flask was sealed and degassed by N_2_ purging for 30 min. Reactions were placed in an ultrasound bath for 4 hours. Then, conversion, number-average molecular weight (*M*_n_), and dispersity (*M*_w_/*M*_n_) were measured in the same way as described above. The polymer was obtained by precipitation in mixture of cooled MeOH/H_2_O (3/1, v/ v) and dried under vacuum at 60 °C. The product was prepared by compression molding at 130 °C.

**Synthesis of PMMA-based mechanoluminescent hybrid materials**

4 mL of methyl methacrylate (MMA, 3.8 g, 37.7 mmol, 400 equiv.), 13.8 μL of EBiB (18.3 mg, 94.1 μmol, 1 equiv.), 0.63 mg of CuBr_2_ (2.8 μmol, 0.03 equiv.), 3.3 mg of TPMA (11.3 μmol, 0.12 equiv.) 400 mg ML, and 4 mL of DMSO were added to a 25 mL Schlenk flask. The reaction flask was sealed and degassed by N_2_ purging for 30 min. Reactions were placed in an ultrasound bath for 14 hours. Then, conversion, number-average molecular weight (*M*_n_), and dispersity (*M*_w_/*M*_n_) were measured in the same way as described above. The polymer was obtained by precipitation in mixture of cooled MeOH/H_2_O (3/1, v/ v) and dried under vacuum at 60 °C. The product was prepared by compression molding at 150 °C.


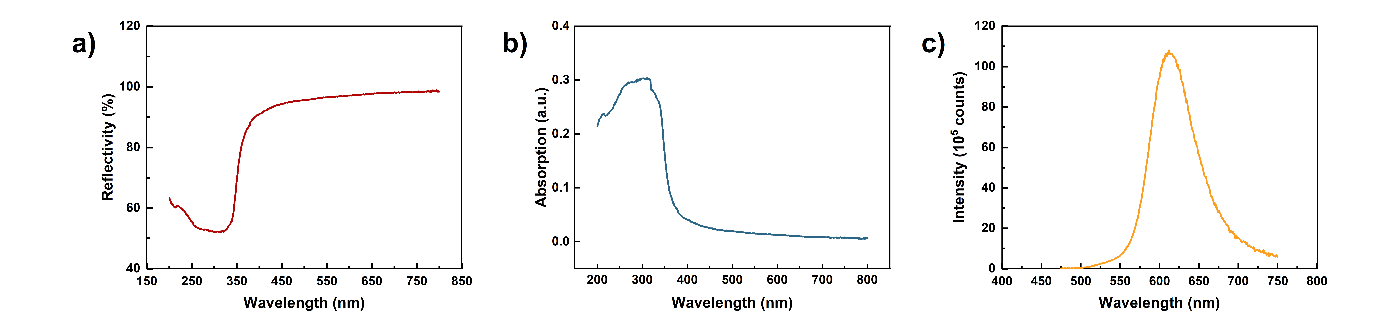


**Figure S1.** (a) Reflectance spectrum of ML powders (CaZnOS-ZnS-SrZnOS); (b) UV-vis absorption spectra of ML powders ; (c) Emission spectrum of ML powders.


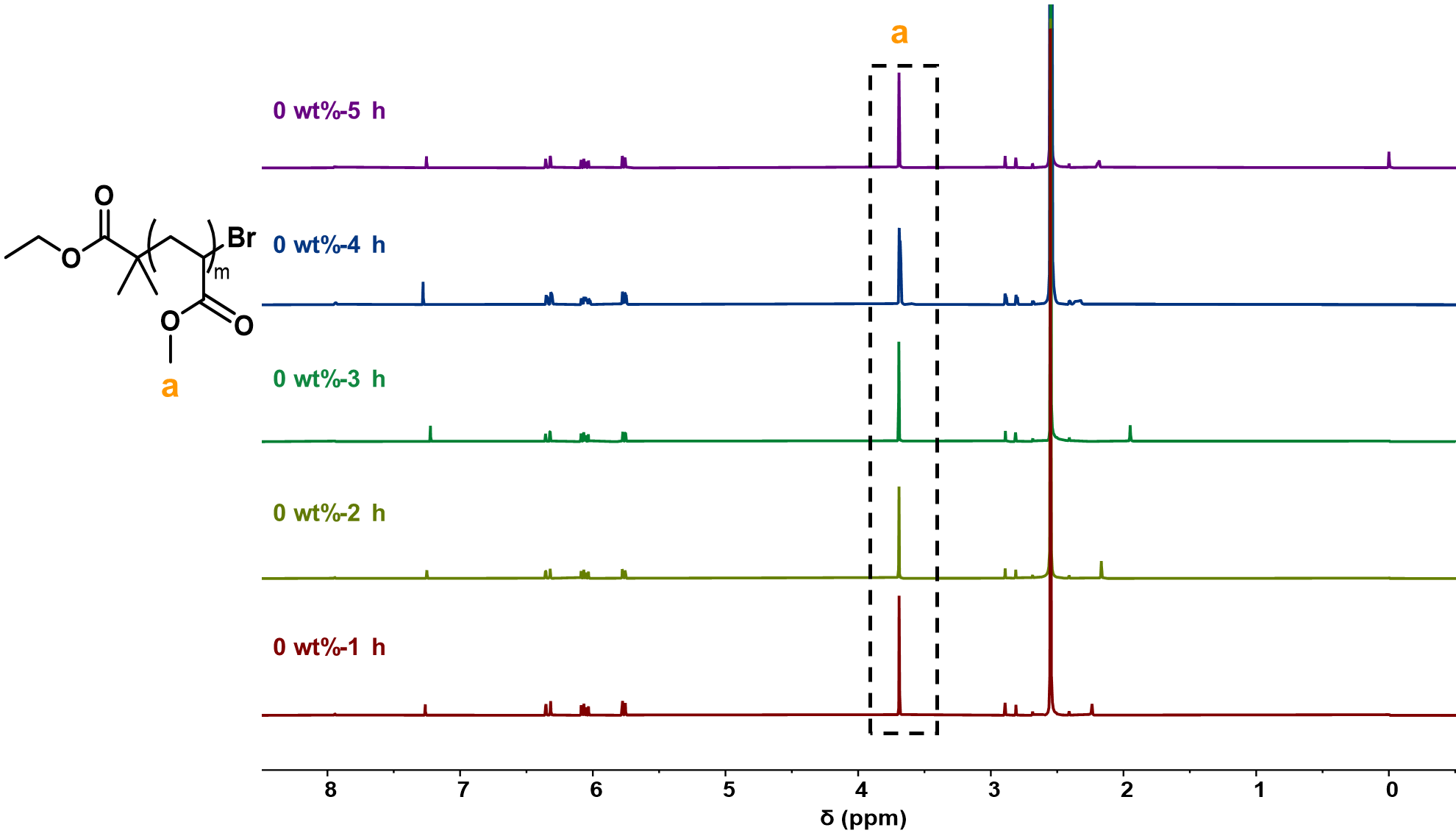


**Figure S2.** Conversion analysis by ^1^H NMR (CDCl_3_) of reaction mixture of Mechano-ATRP of MA under 0 wt% ML powders.


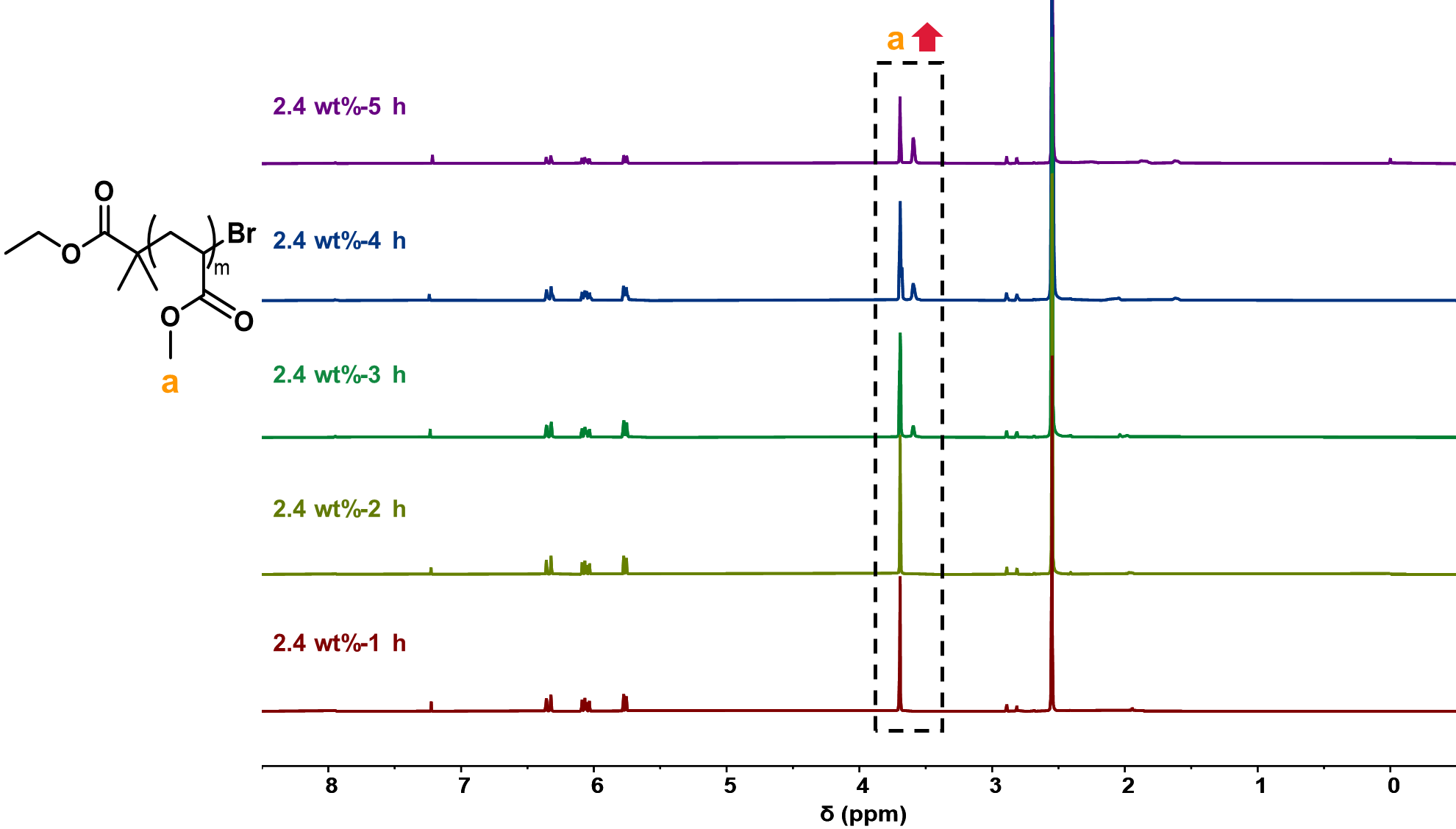


**Figure S3.** Conversion analysis by ^1^H NMR (CDCl_3_) of reaction mixture of Mechano-ATRP of MA under 2.4 wt% ML powders.


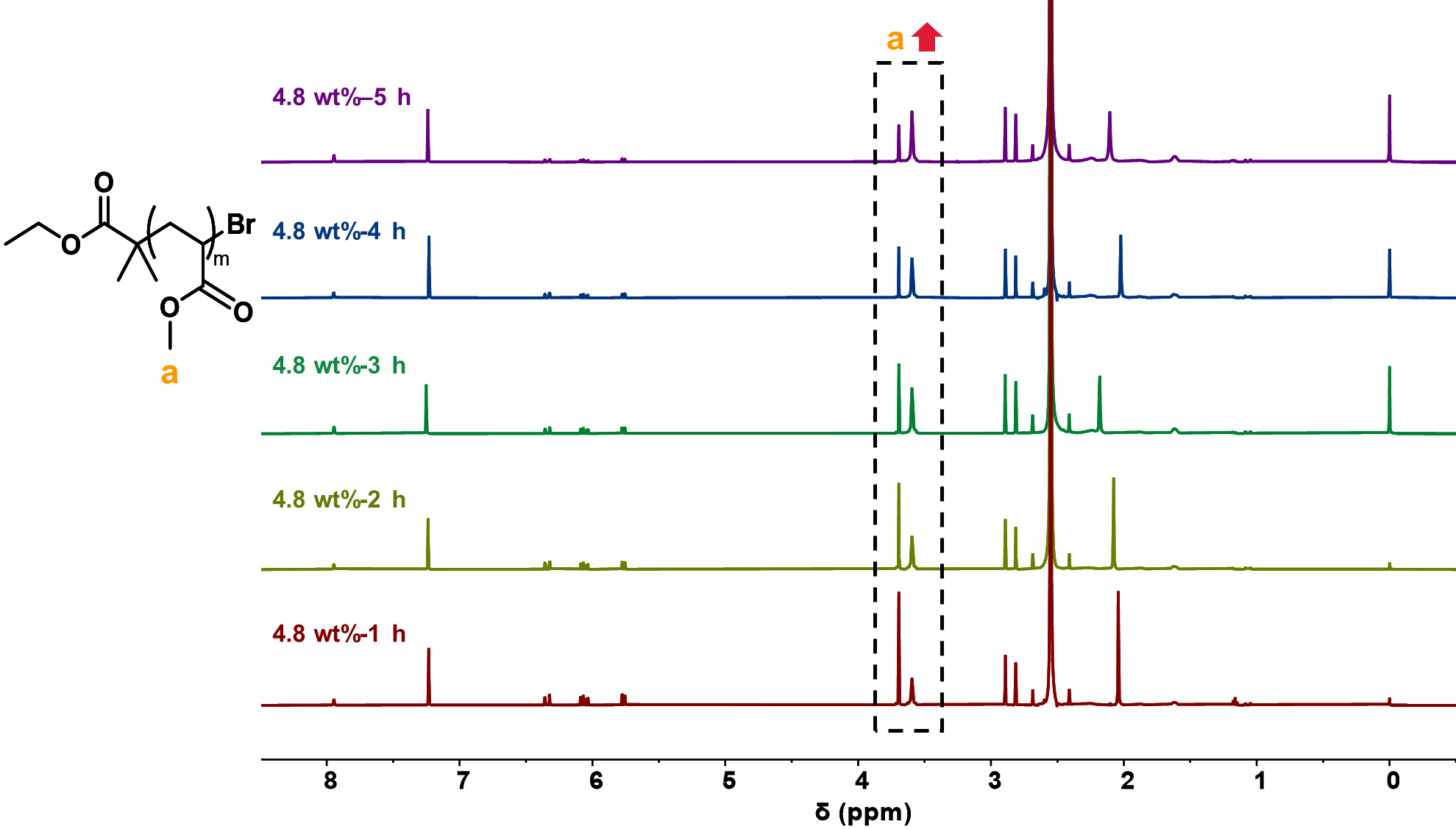


**Figure S4.** Conversion analysis by ^1^H NMR (CDCl_3_) of reaction mixture of Mechano-ATRP of MA under 4.8 wt% ML powders.


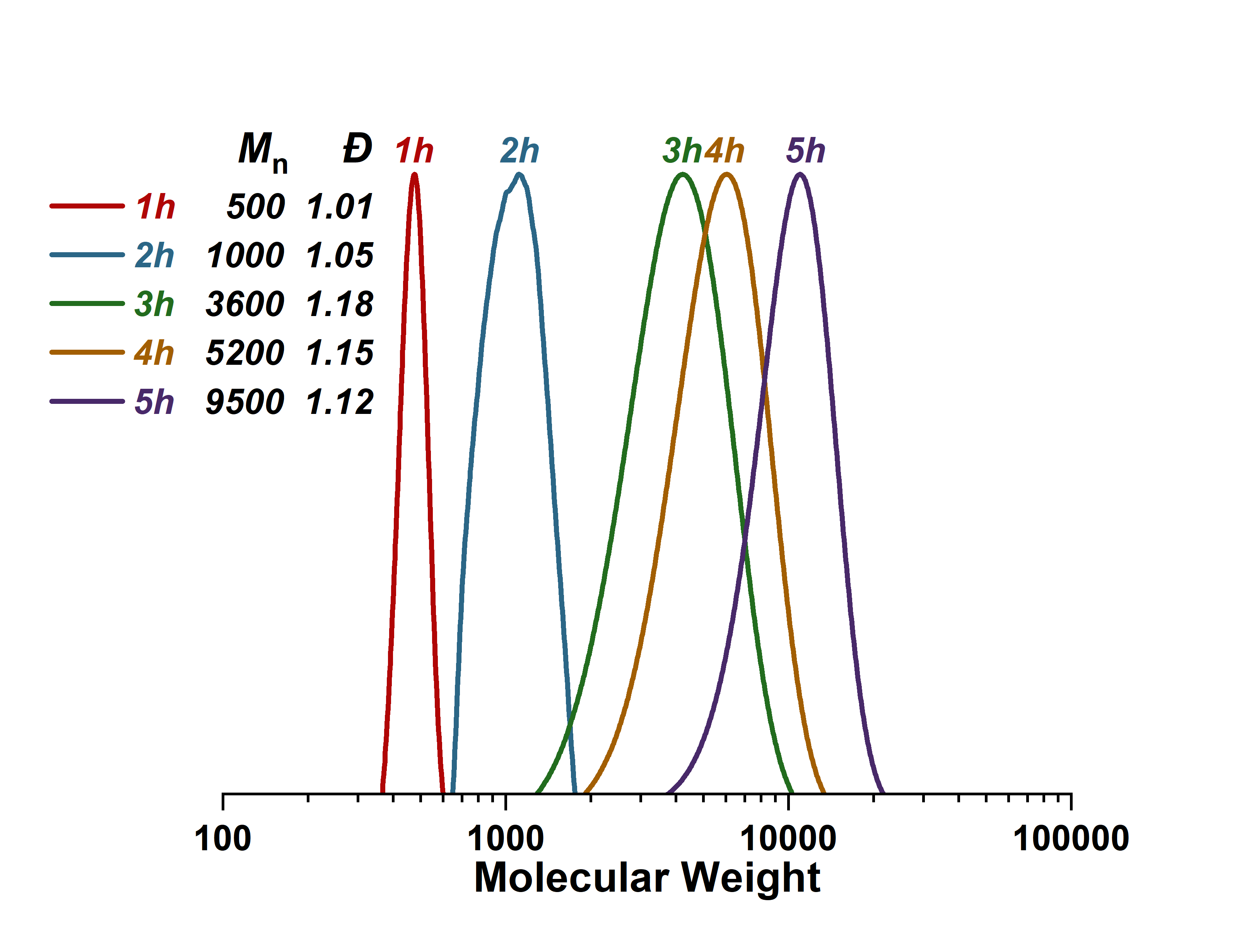


**Figure S5.** GPC traces of Mechano-ATRP of MA with 2.4 wt% ML powders.


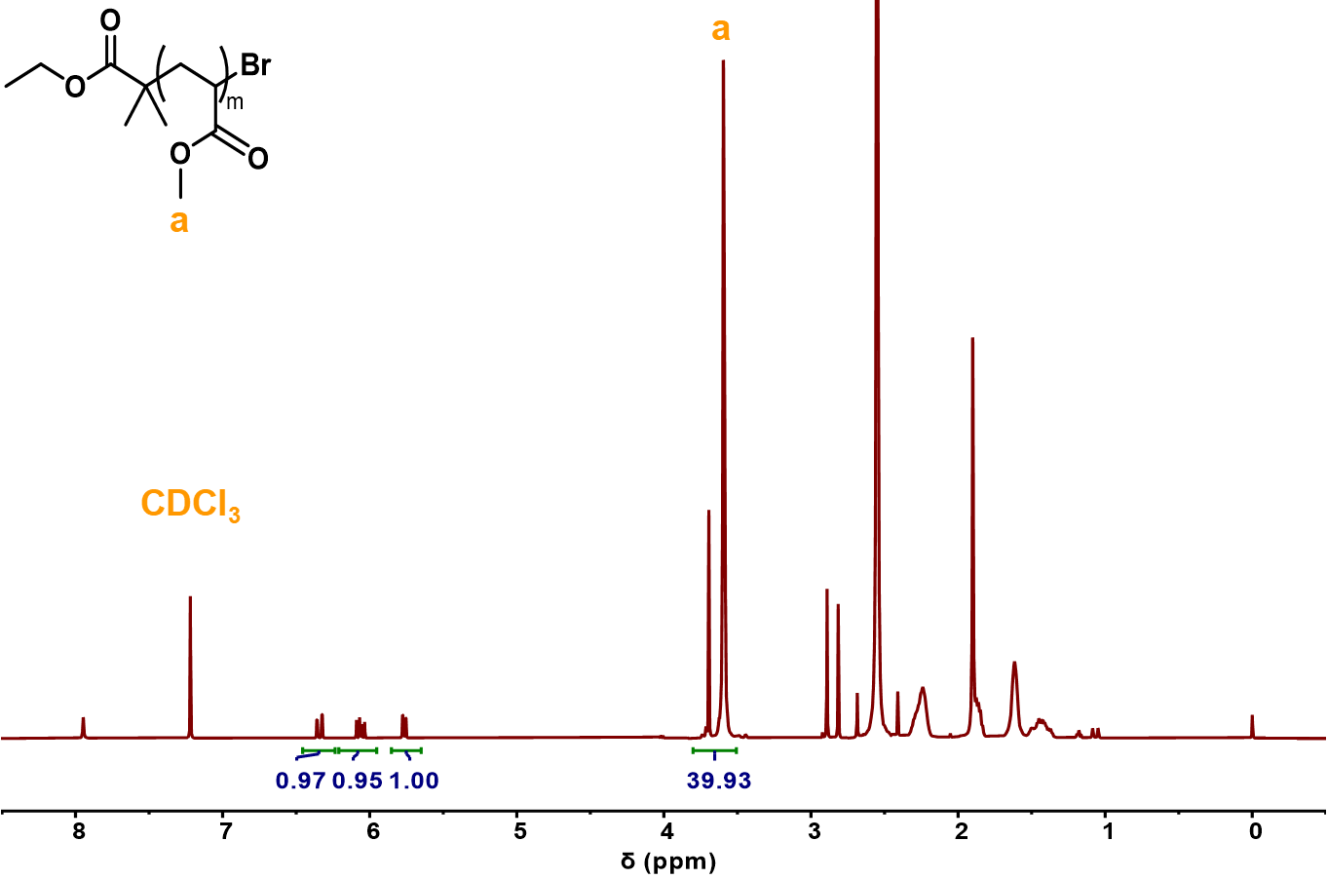


**Figure S6.** Conversion analysis by ^1^H NMR (CDCl_3_) of reaction mixture of Mechano-ATRP of MA with *DP*_T_ = 200 (entry 2).


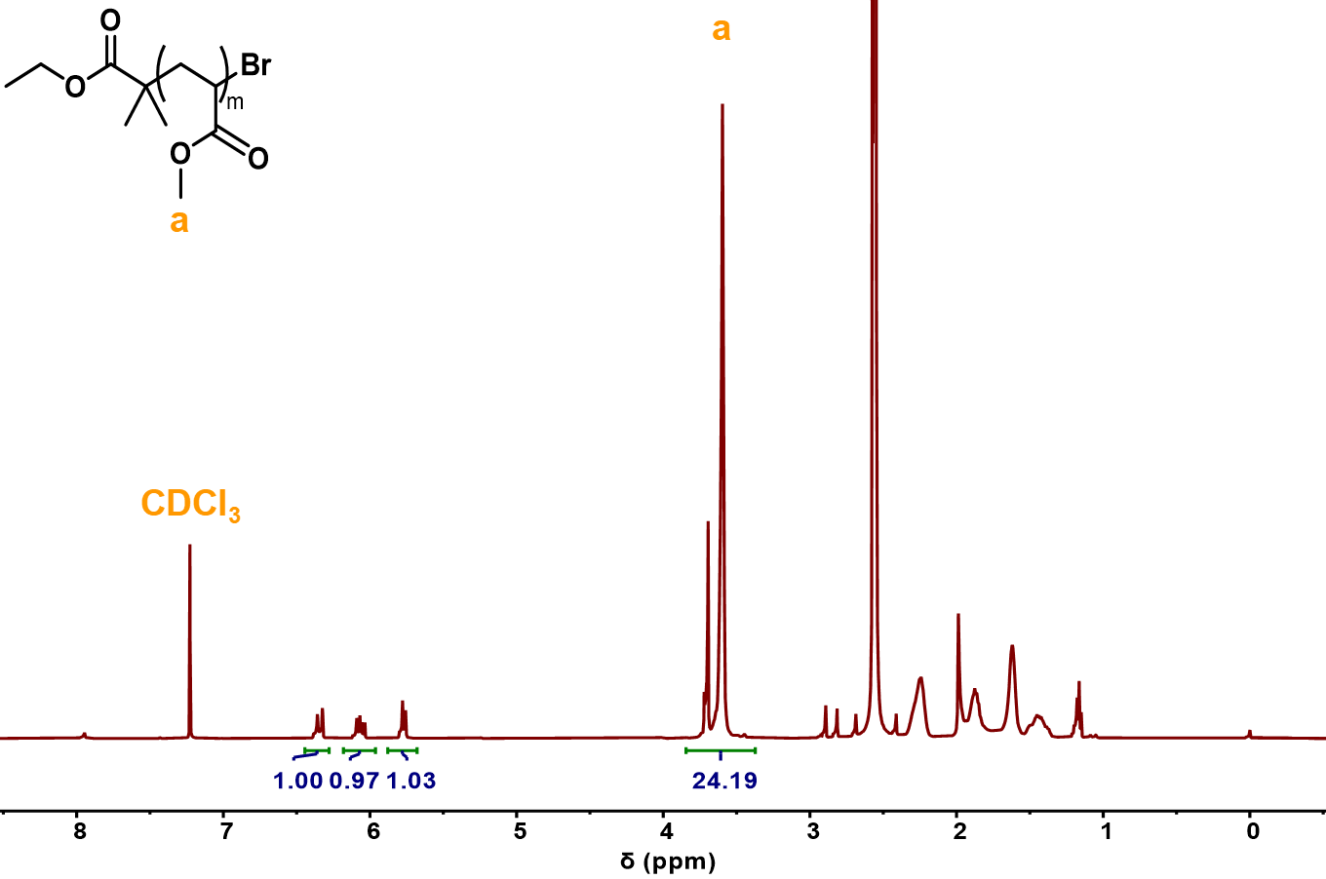


**Figure S7.** Conversion analysis by ^1^H NMR (CDCl_3_) of reaction mixture of Mechano-ATRP of MA with *DP*_T_ = 400 (entry 3).


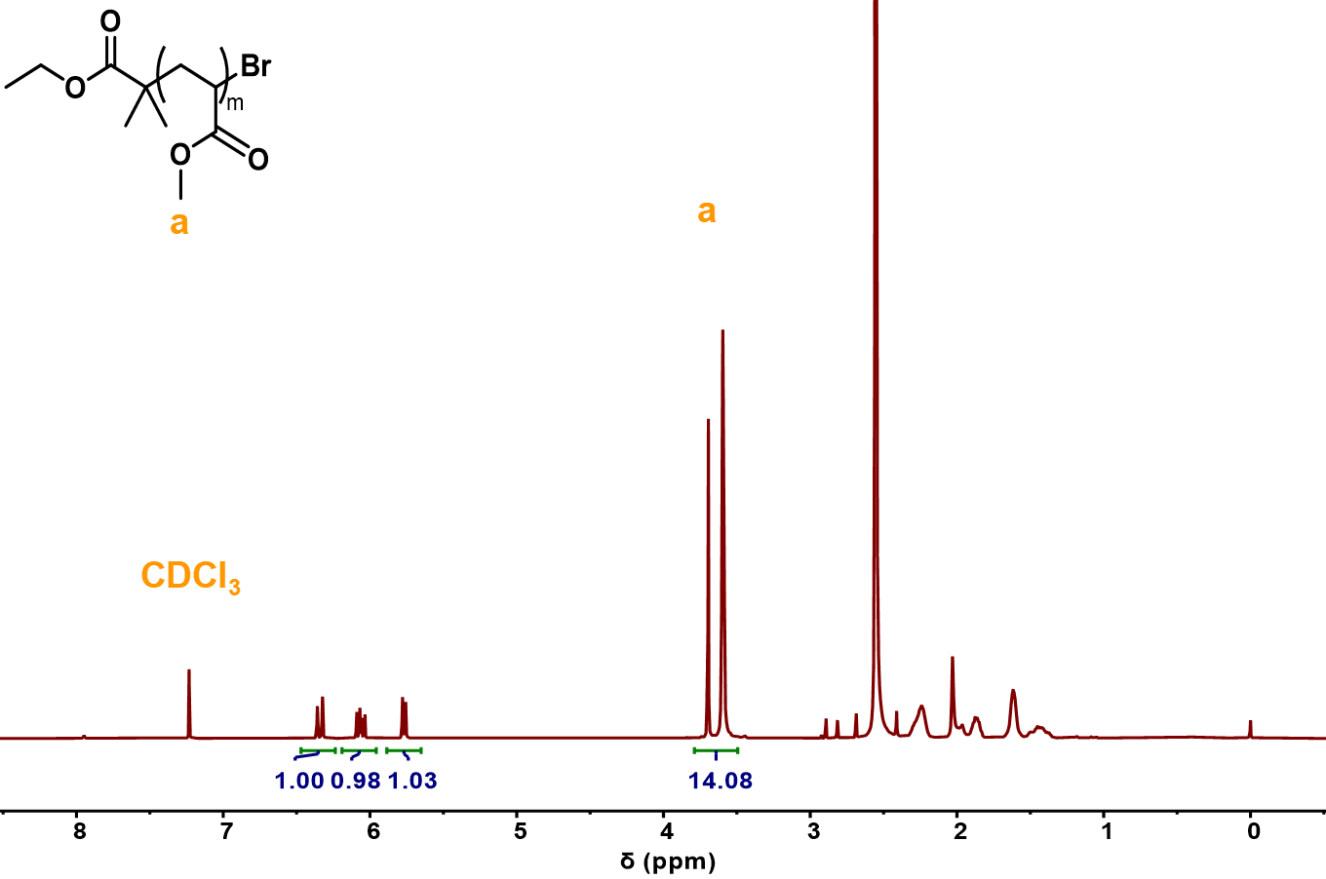


**Figure S8.** Conversion analysis by ^1^H NMR (CDCl_3_) of reaction mixture of Mechano-ATRP of MA with *DP*_T_ = 800 (entry 4).


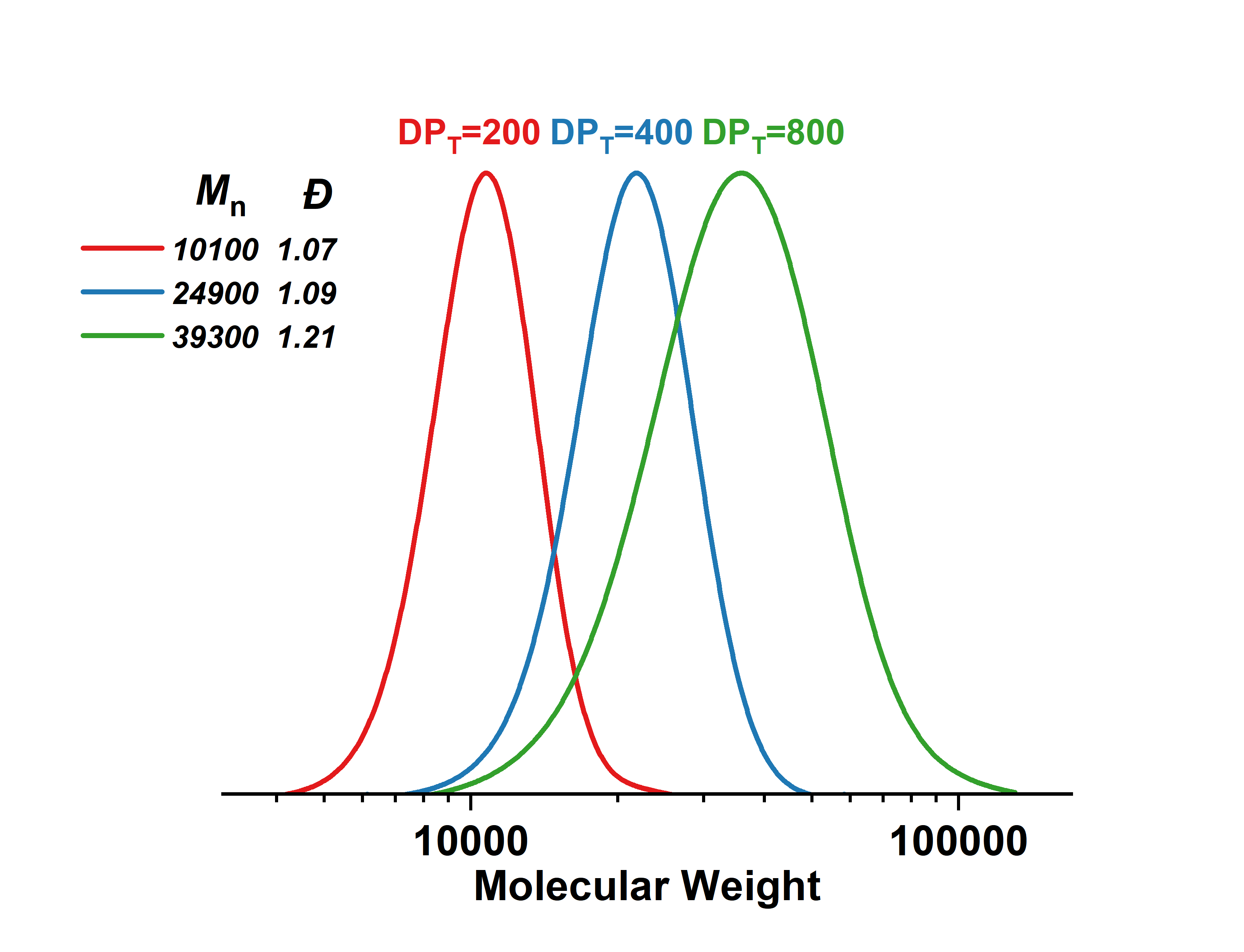


**Figure S9.** GPC traces of Mechano-ATRP of MA with different *DP*_T_.


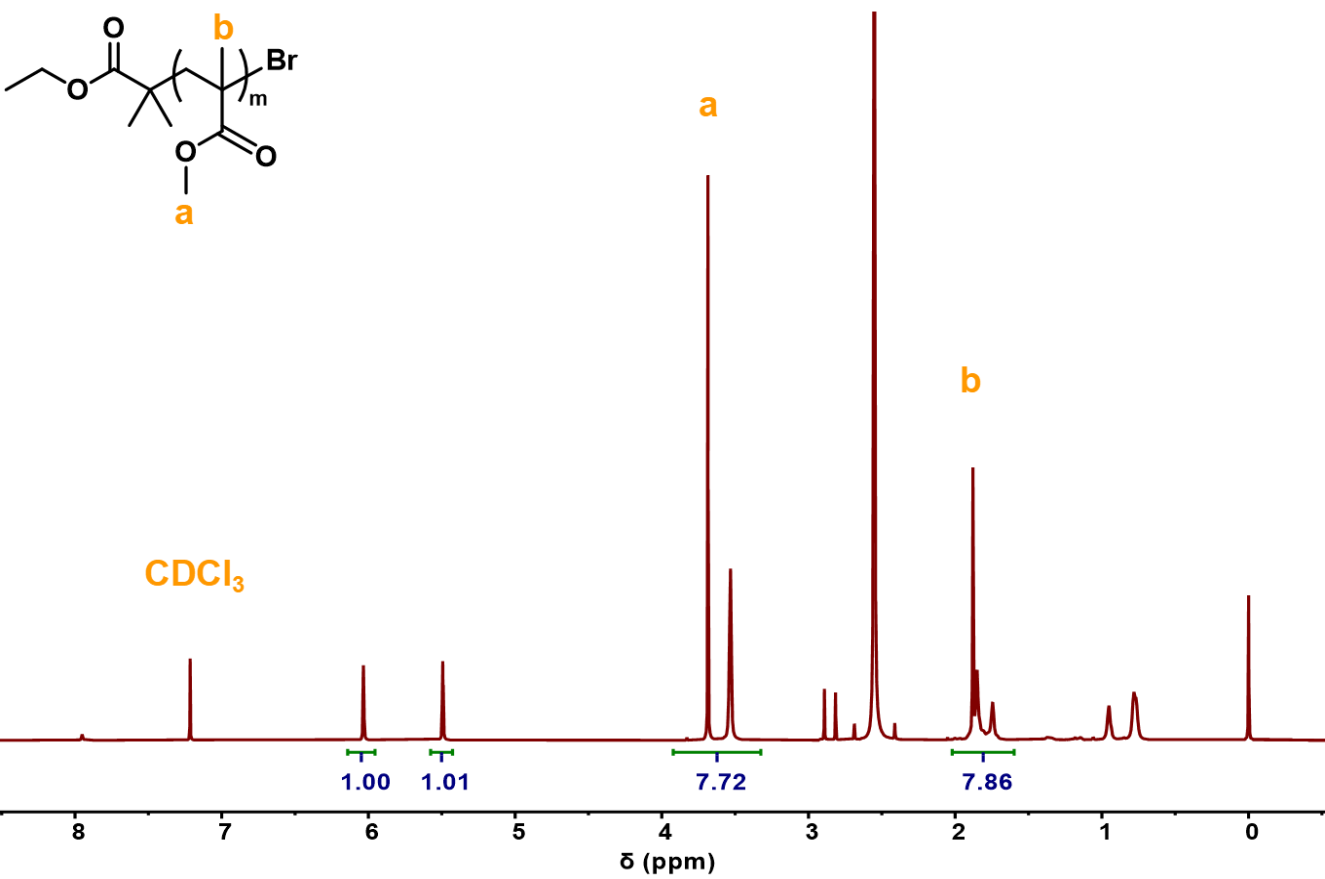


**Figure S10.** Conversion analysis by ^1^H NMR (CDCl_3_) of reaction mixture of Mechano-ATRP of MMA with *DP*_T_ = 200 (entry 5).


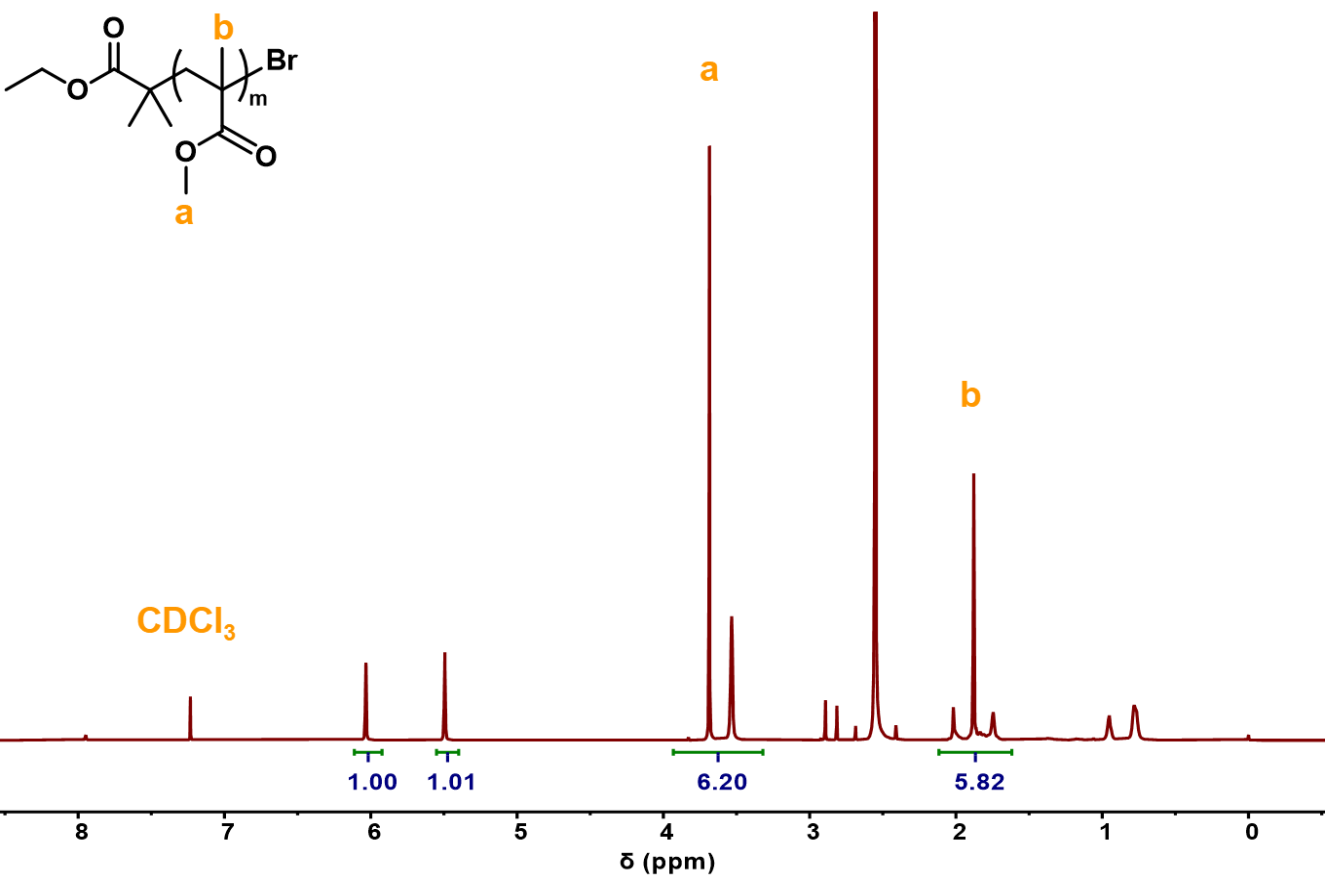


**Figure S11.** Conversion analysis by ^1^H NMR (CDCl_3_) of reaction mixture of Mechano-ATRP of MMA with *DP*_T_ = 400 (entry 6).


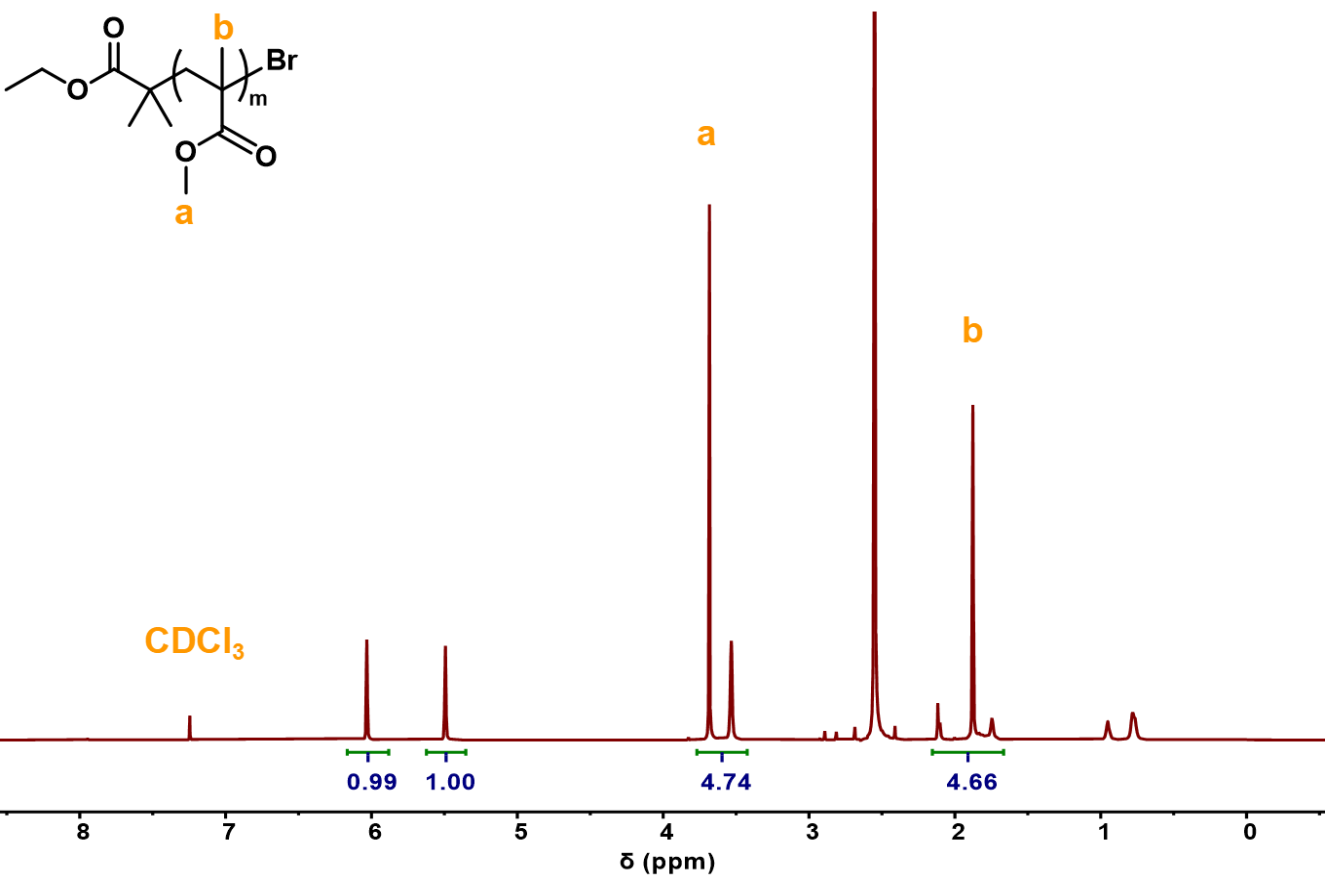


**Figure S12.** Conversion analysis by ^1^H NMR (CDCl_3_) of reaction mixture of Mechano-ATRP of MMA with *DP*_T_ = 800 (entry 7).


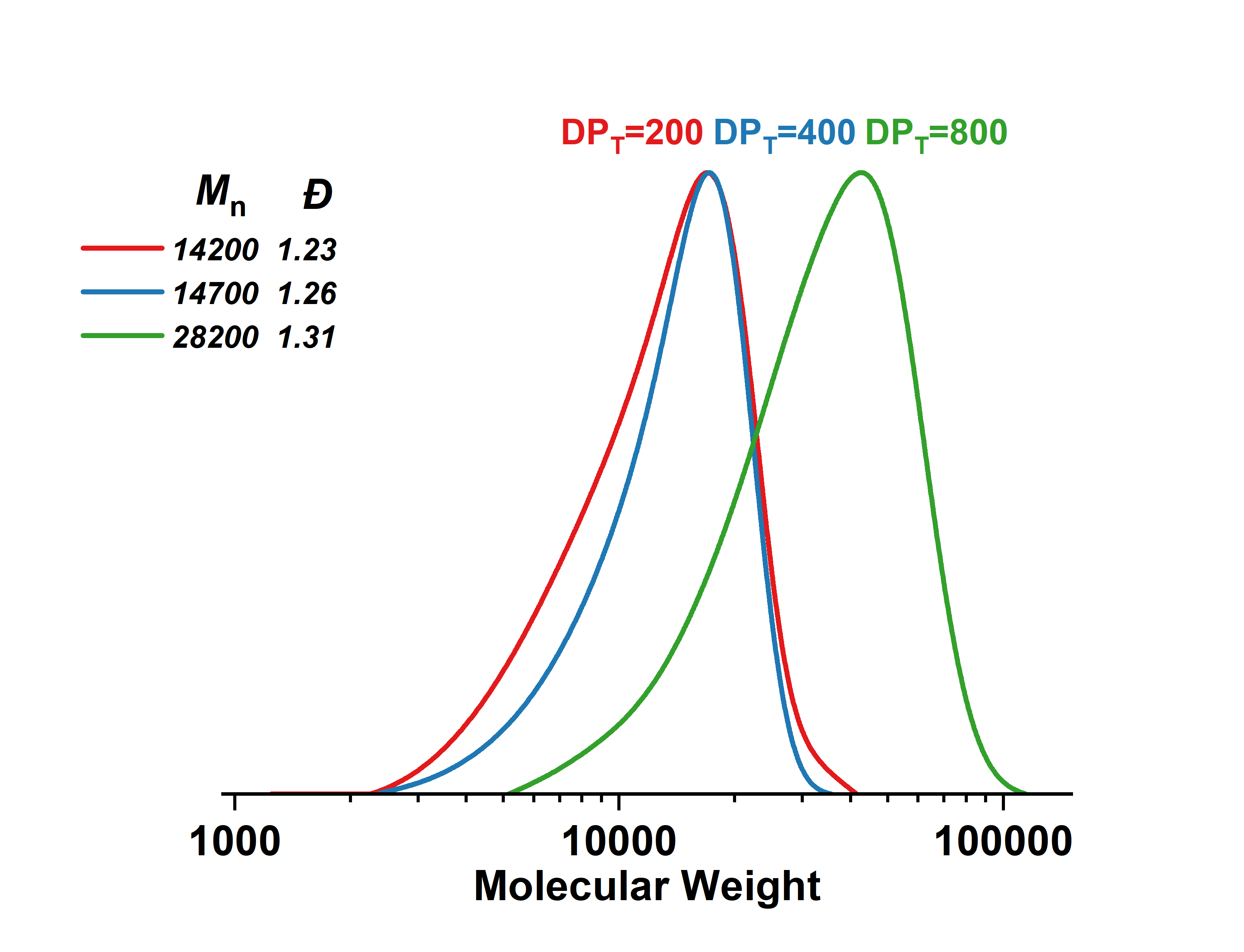


**Figure S13.** GPC traces of Mechano-ATRP of MMA with different *DP*_T_.


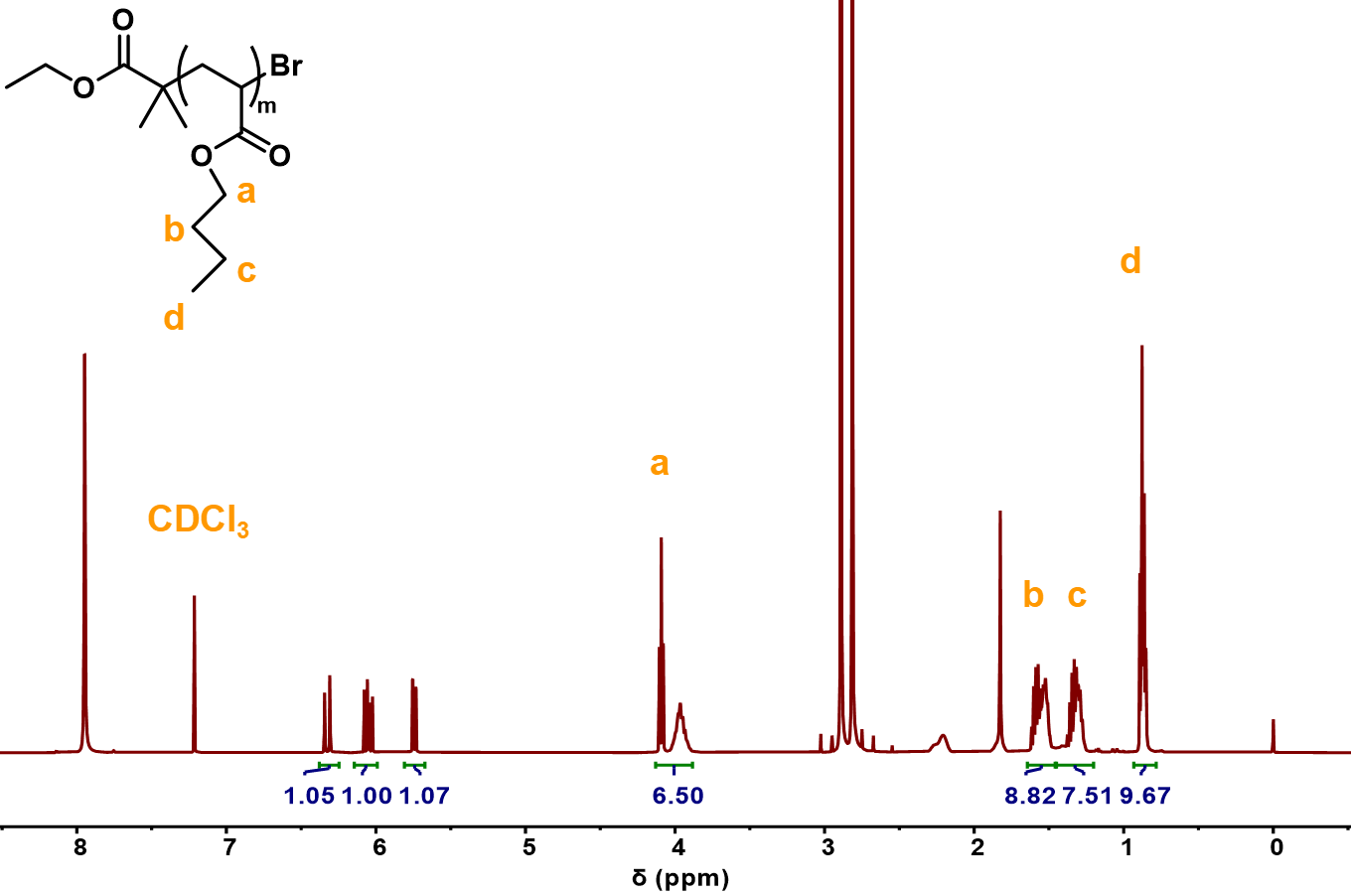

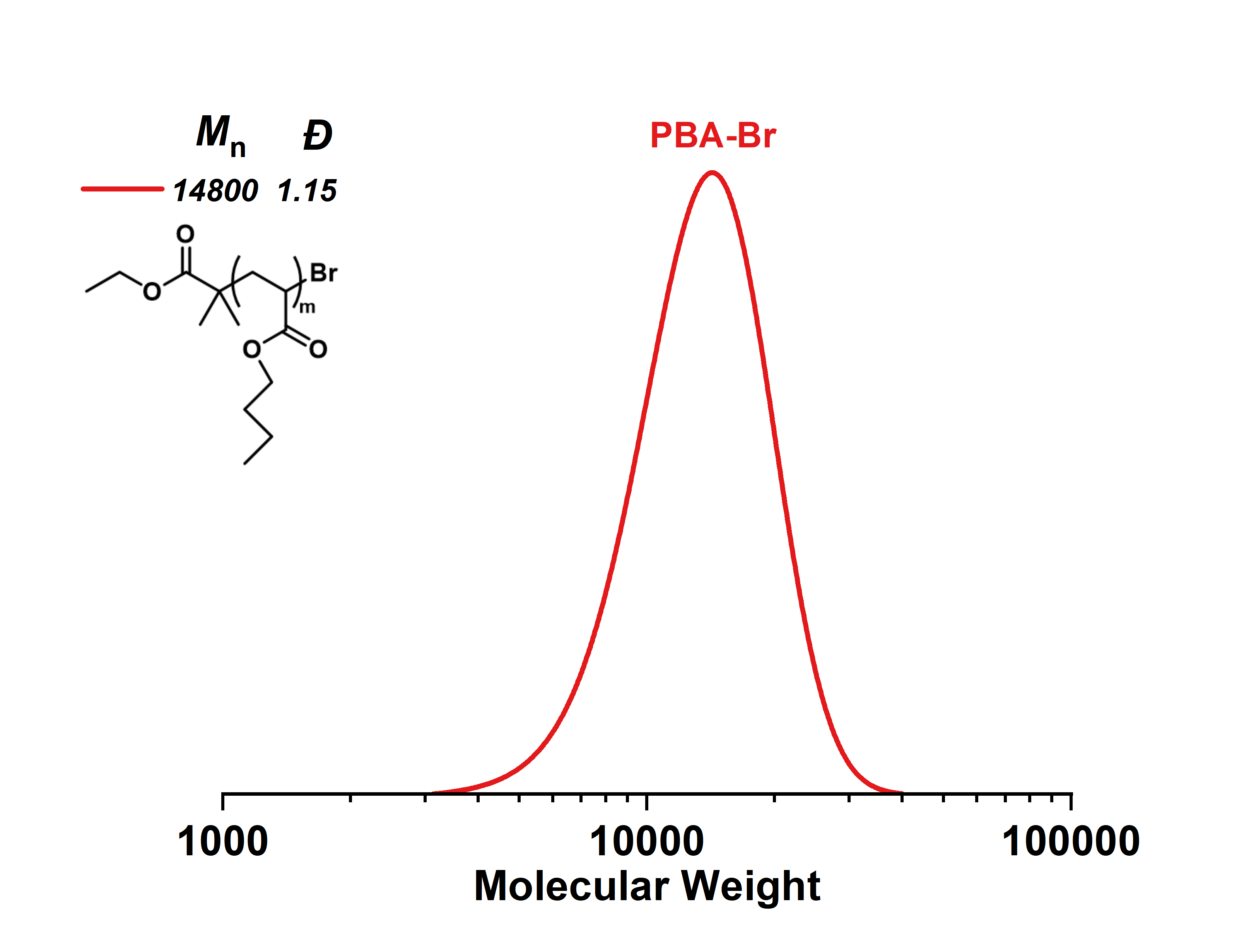


**Figure S14.** Conversion analysis by ^1^H NMR (CDCl_3_) of reaction mixture of Mechano-ATRP of BA with *DP*_T_ = 200 and the GPC trace (entry 8).


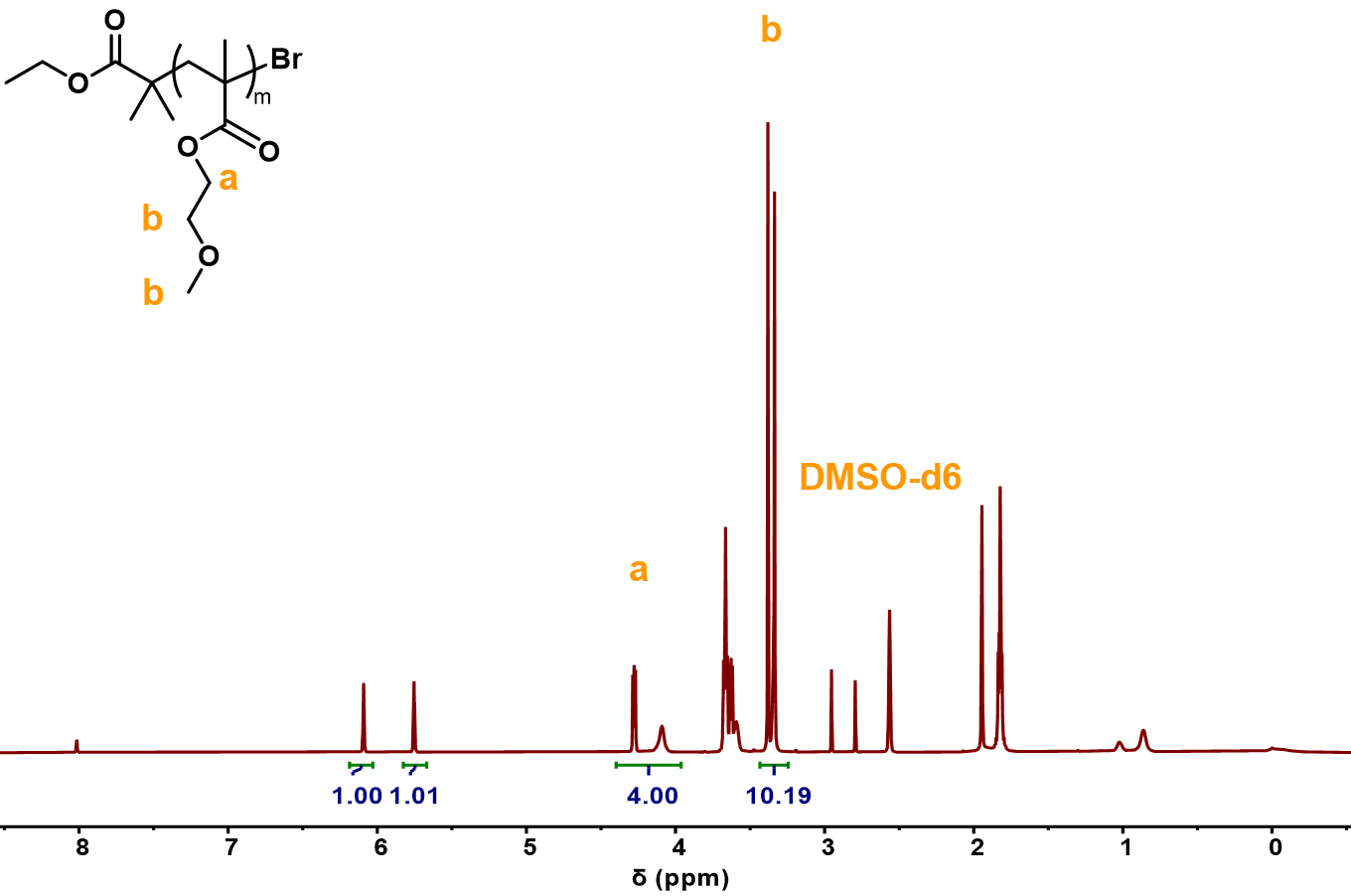

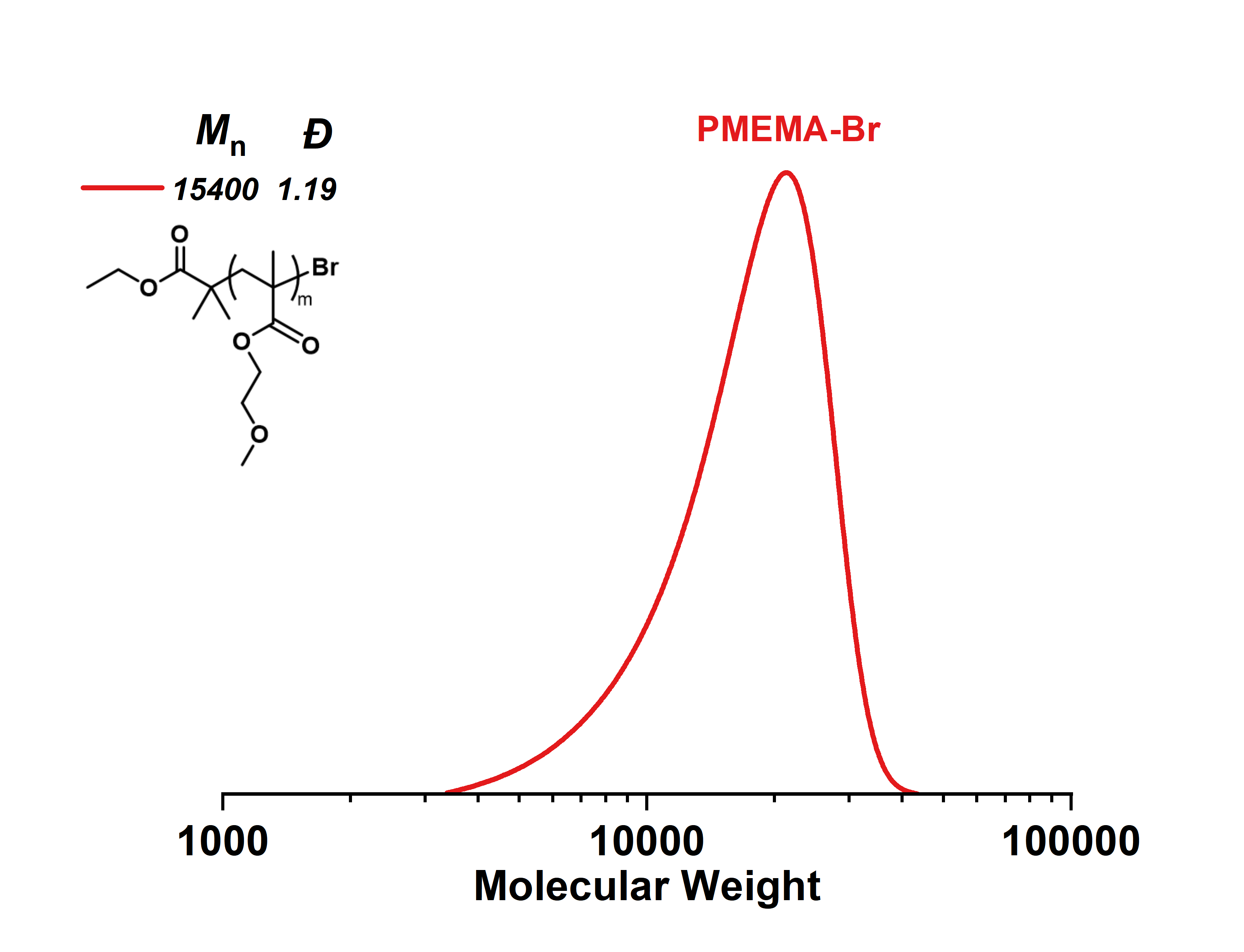


**Figure S15.** Conversion analysis by ^1^H NMR (CDCl_3_) of reaction mixture of Mechano-ATRP of MEMA with *DP*_T_ = 200 and the GPC trace (entry 9).


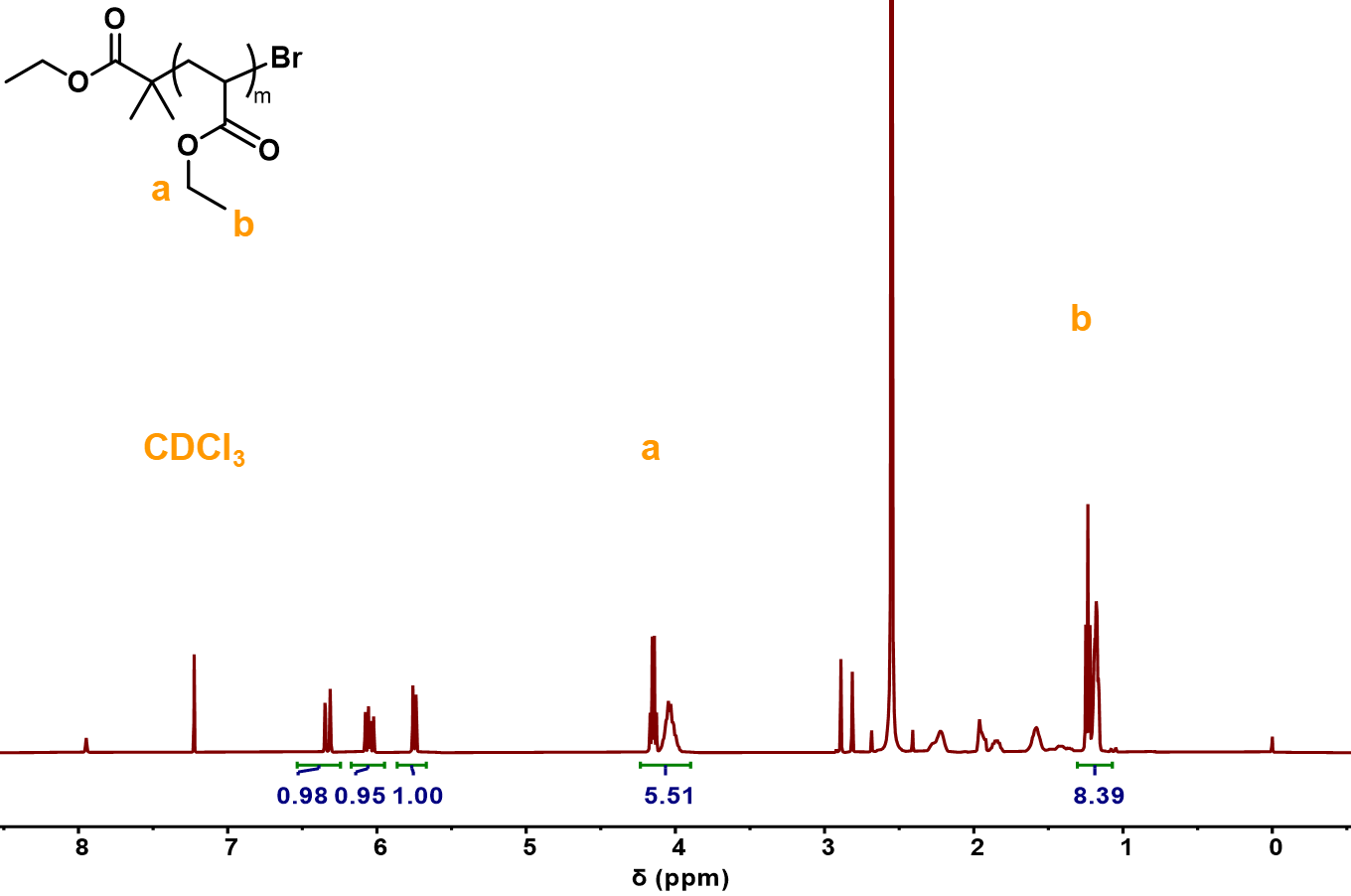

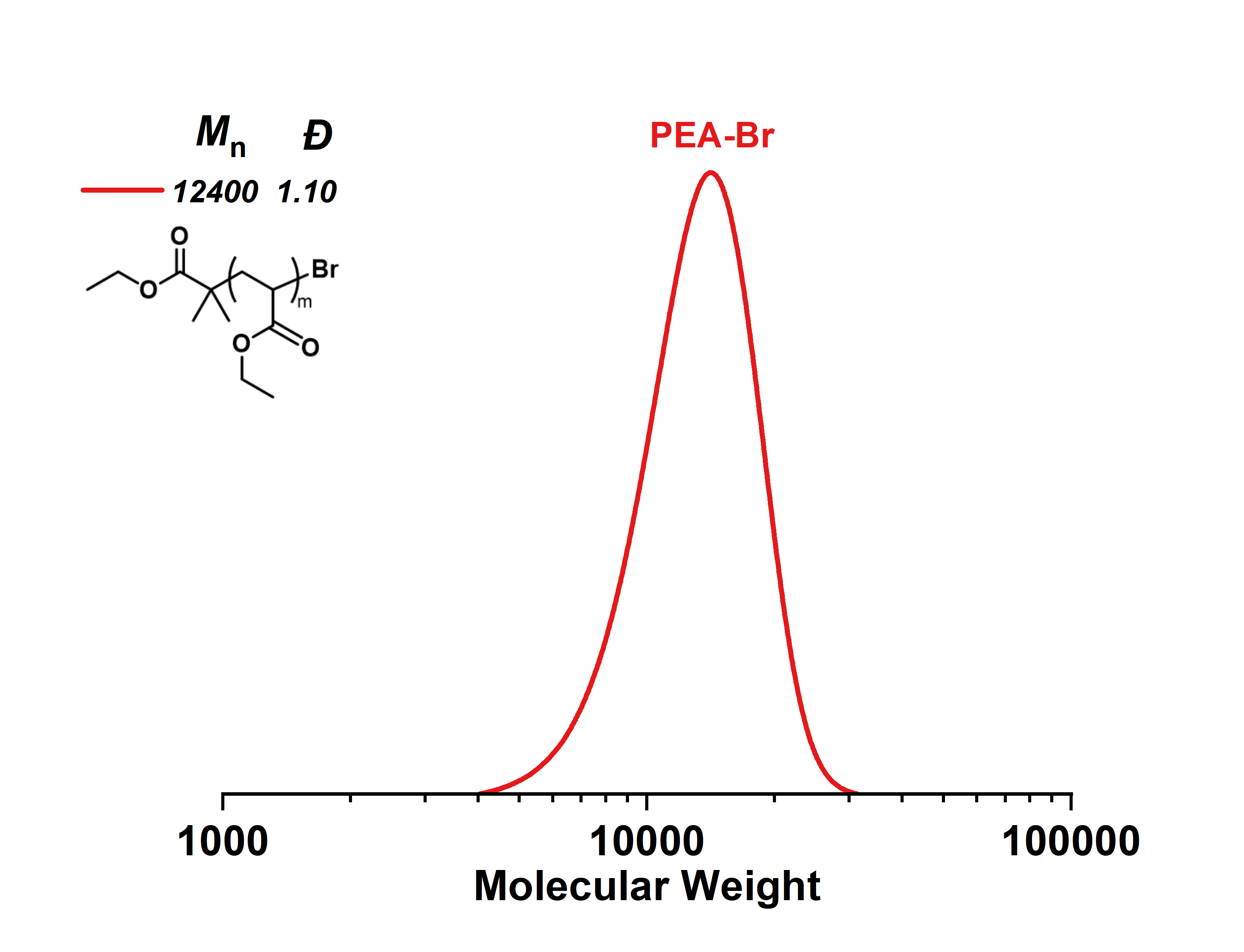


**Figure S16.** Conversion analysis by ^1^H NMR (CDCl_3_) of reaction mixture of Mechano-ATRP of EA with *DP*_T_ = 200 and the GPC trace (entry 10).


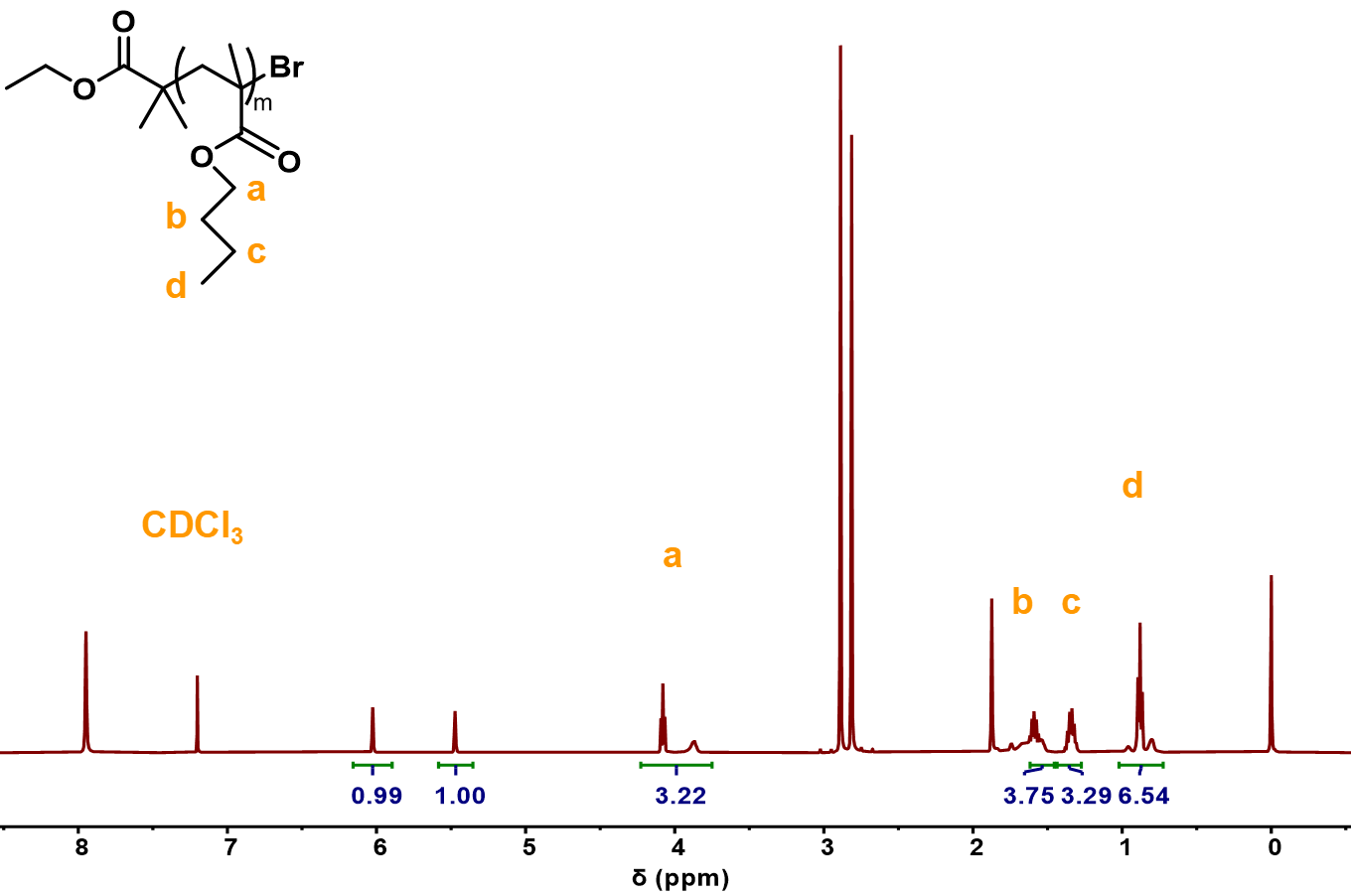

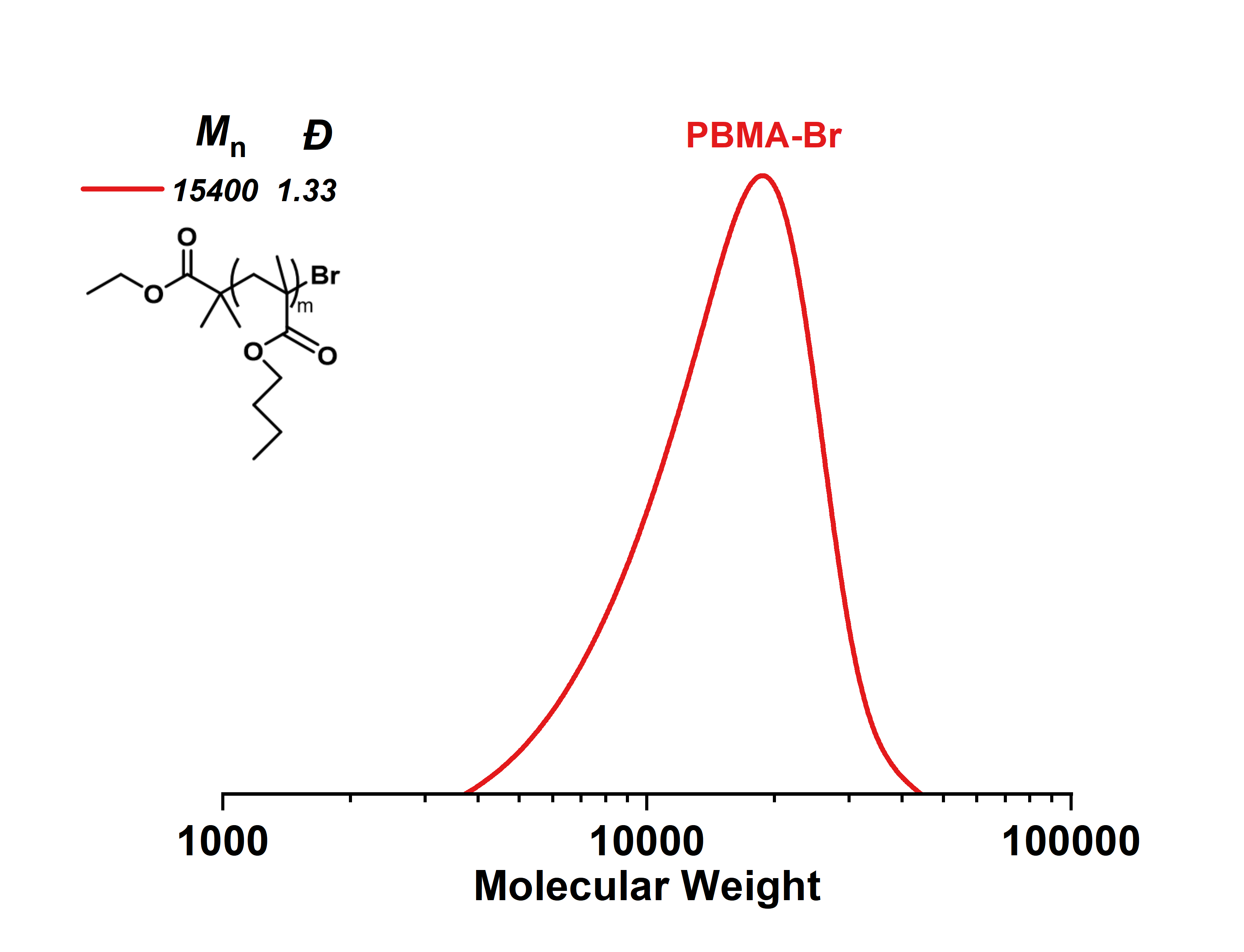


**Figure S17.** Conversion analysis by ^1^H NMR (CDCl_3_) of reaction mixture of Mechano-ATRP of BMA with *DP*_T_ = 200 and the GPC trace (entry 11).


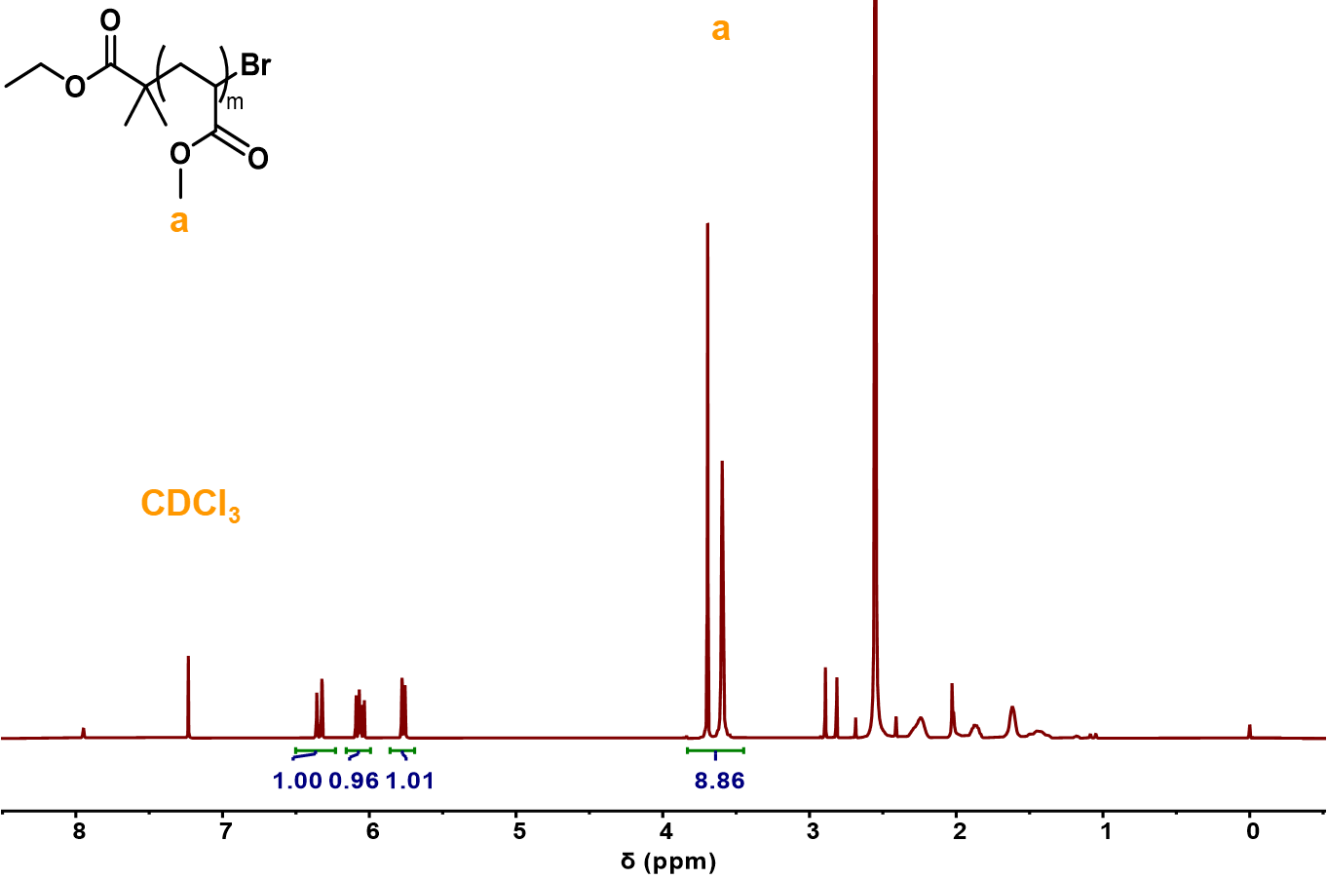


**Figure S18.** Conversion analysis by ^1^H NMR (CDCl_3_) of PMA-Br.


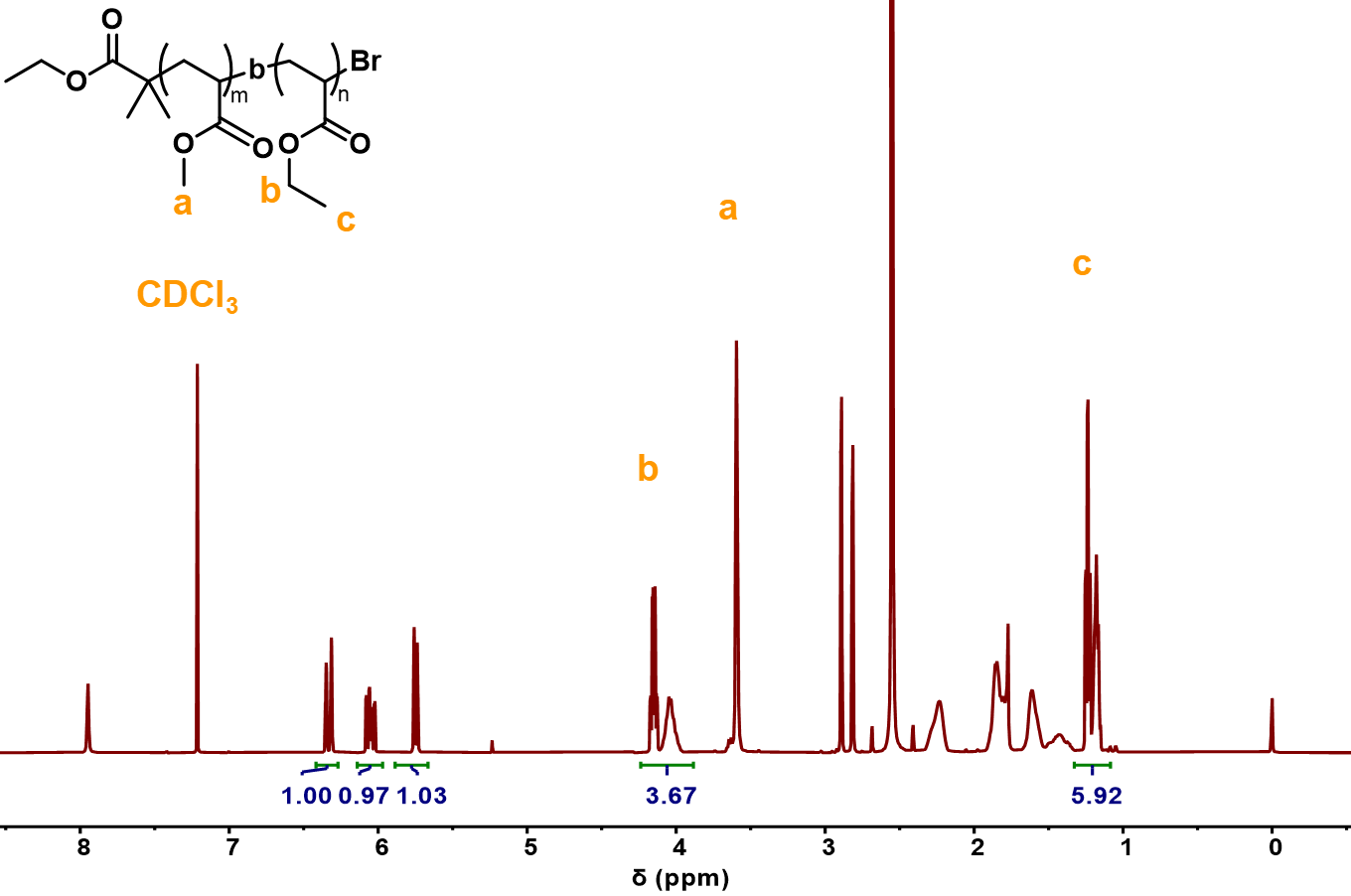


**Figure S19.** Conversion analysis by ^1^H NMR (CDCl_3_) of PMA-*b*-PEA-Br.

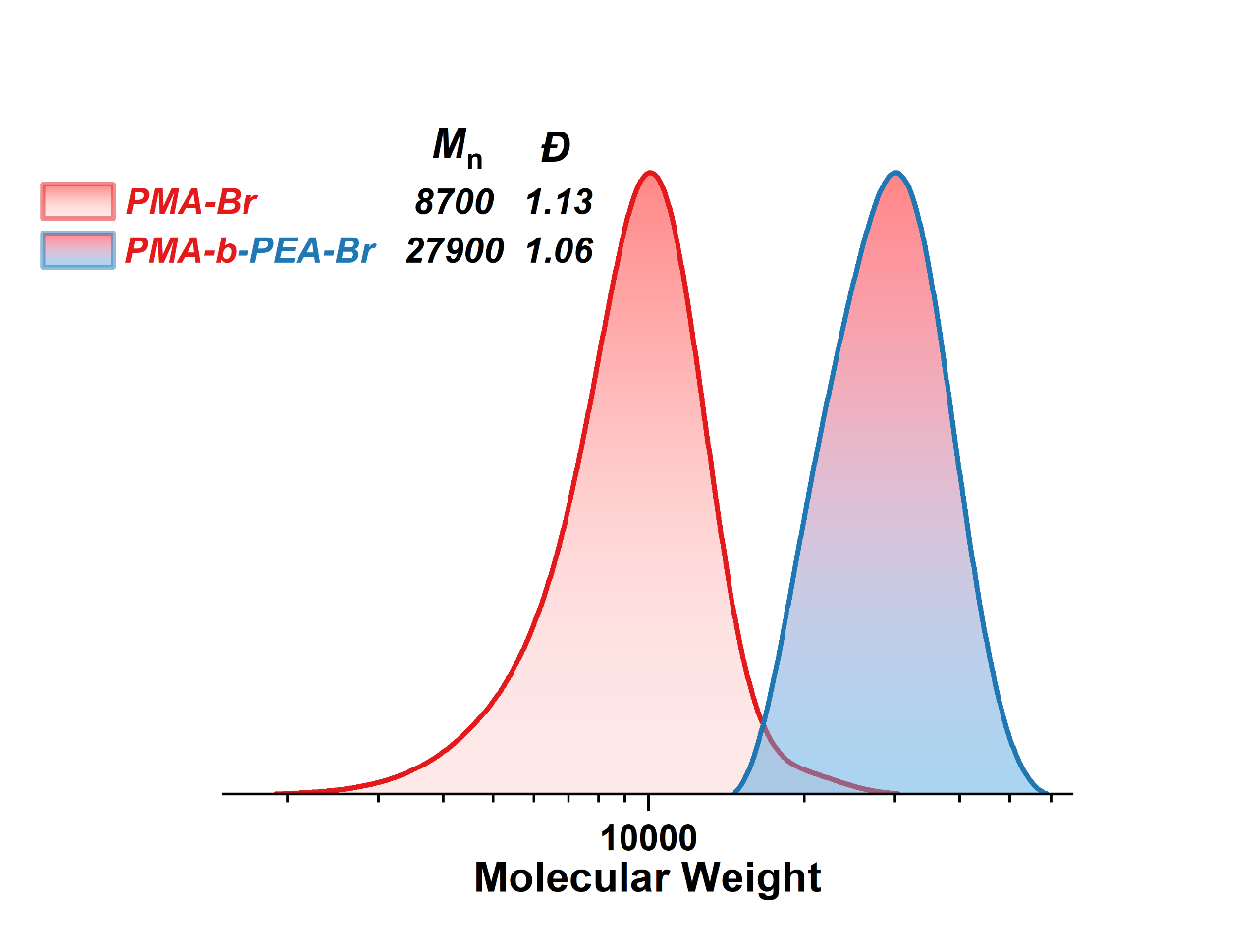


**Figure S20.**GPC traces of PMA-Br (red) and PMA-*b*-PEA-Br (blue).


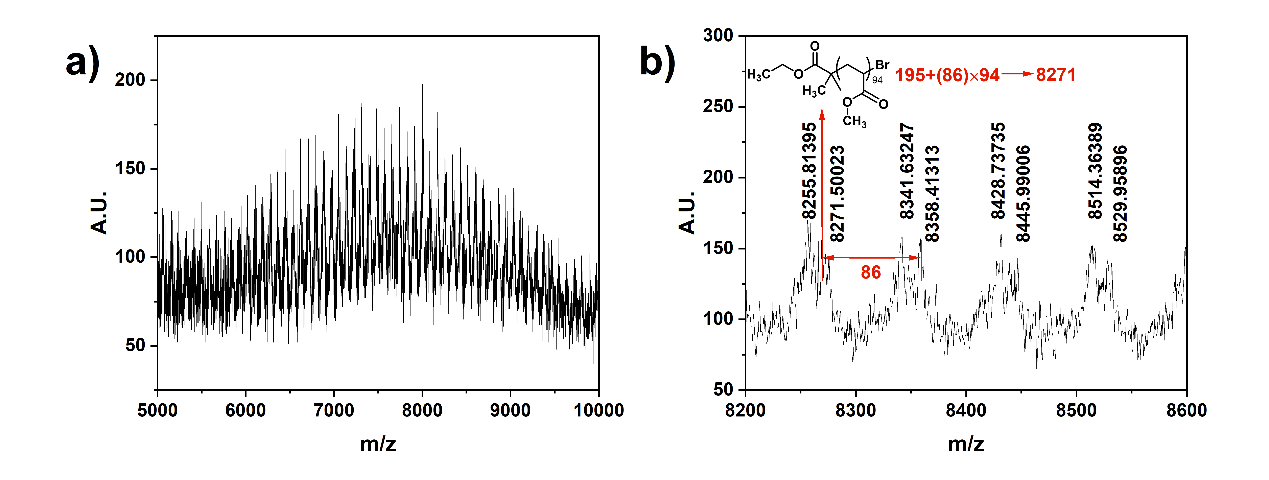


**Figure S21.** (a) The MALDI-TOF spectrum of the poly(methyl acrylate) (PMA); (b) Magnification of a region in the MALDI-TOF-MS spectrum.


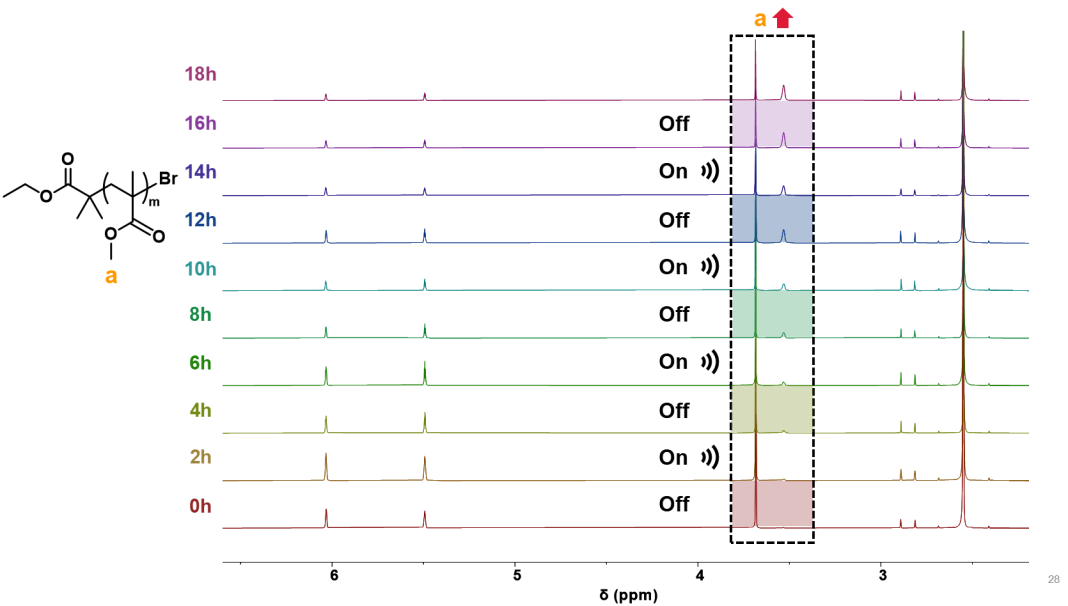


**Figure S22.** Conversion analysis by ^1^H NMR (CDCl_3_) of MMA of ***ON-OFF***.


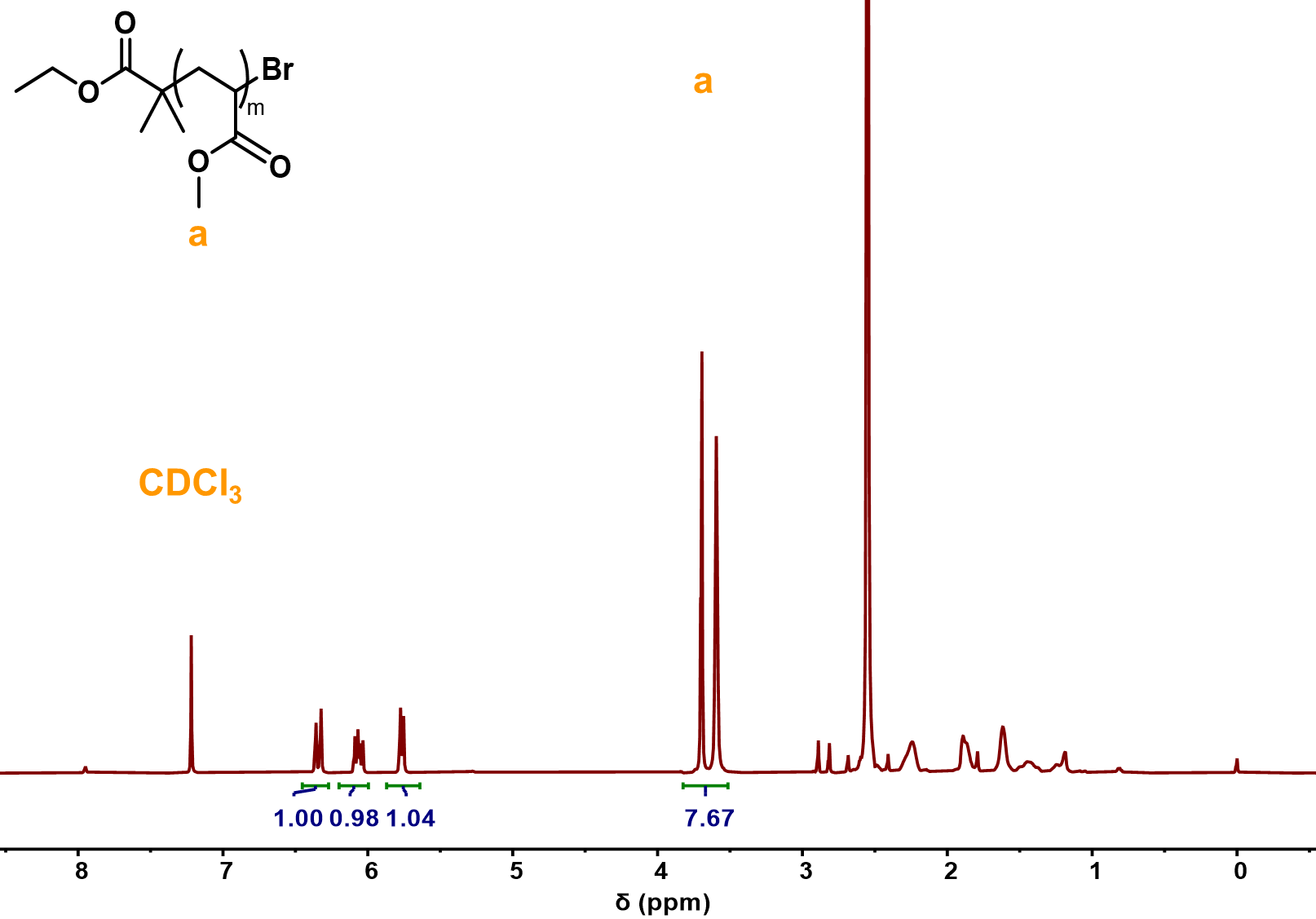


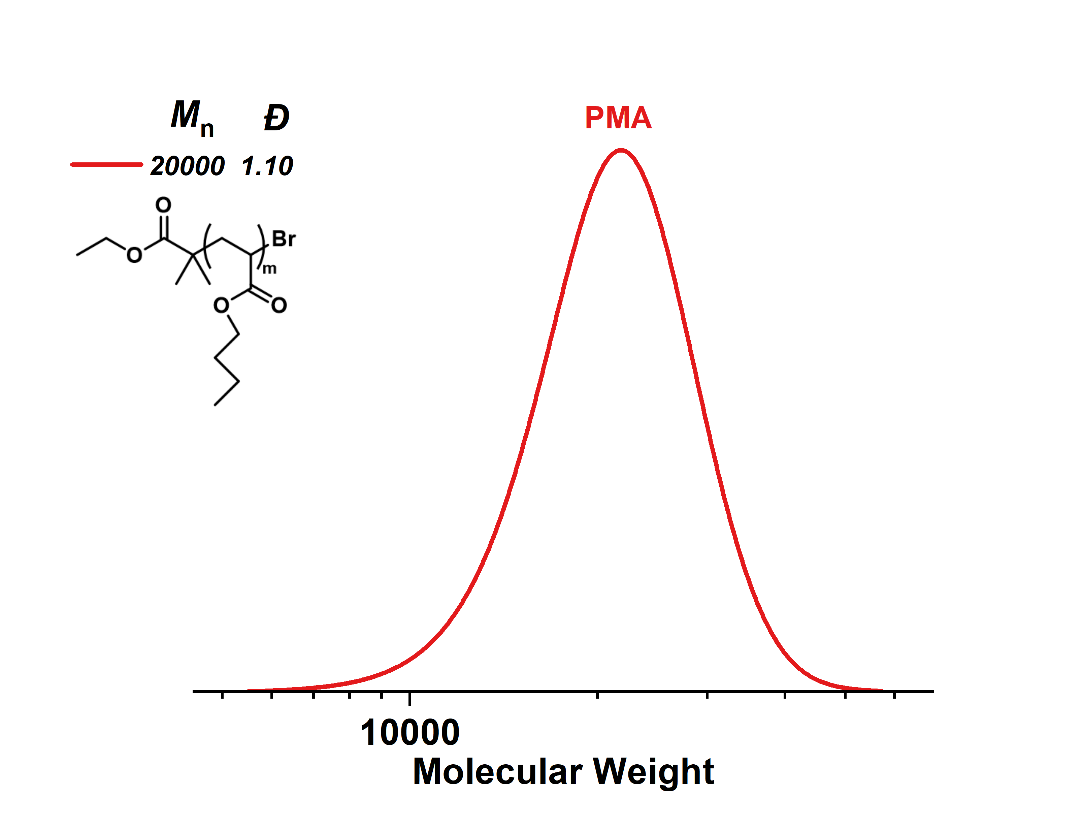


**Figure S23.** Conversion analysis by ^1^H NMR (CDCl_3_) of PMA of mechanoluminescent hybrid materials and the GPC trace.


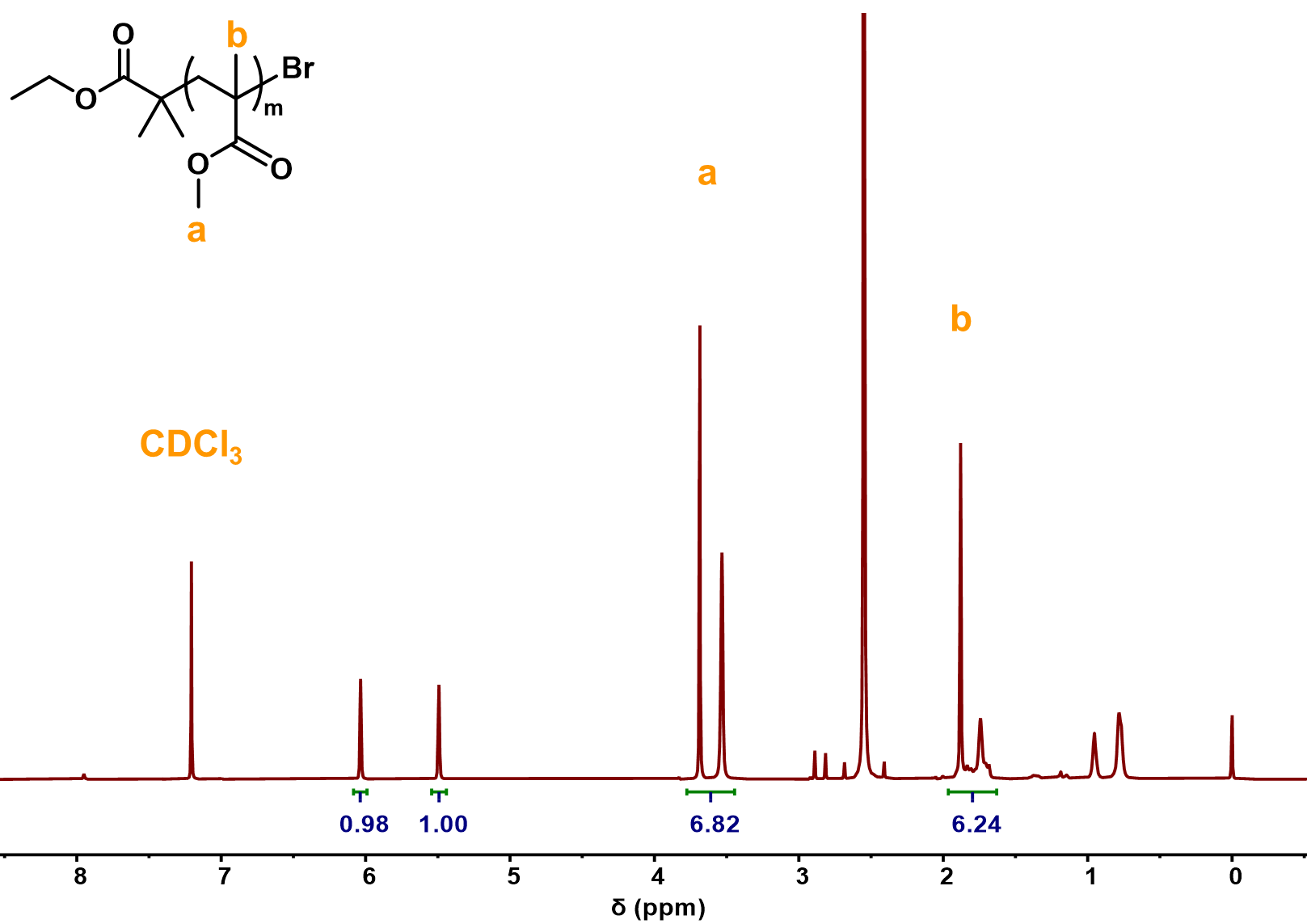


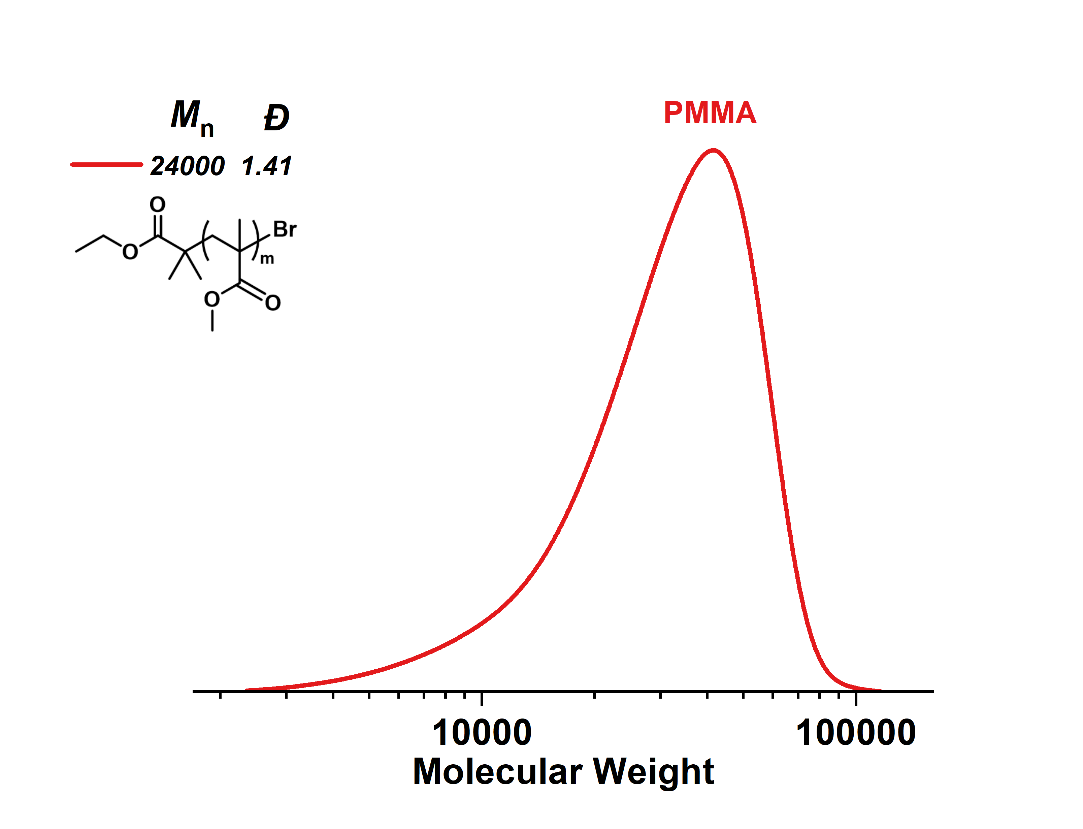


**Figure S24.** Conversion analysis by ^1^H NMR (CDCl_3_) of PMMA of mechanoluminescent hybrid materials and the GPC trace.


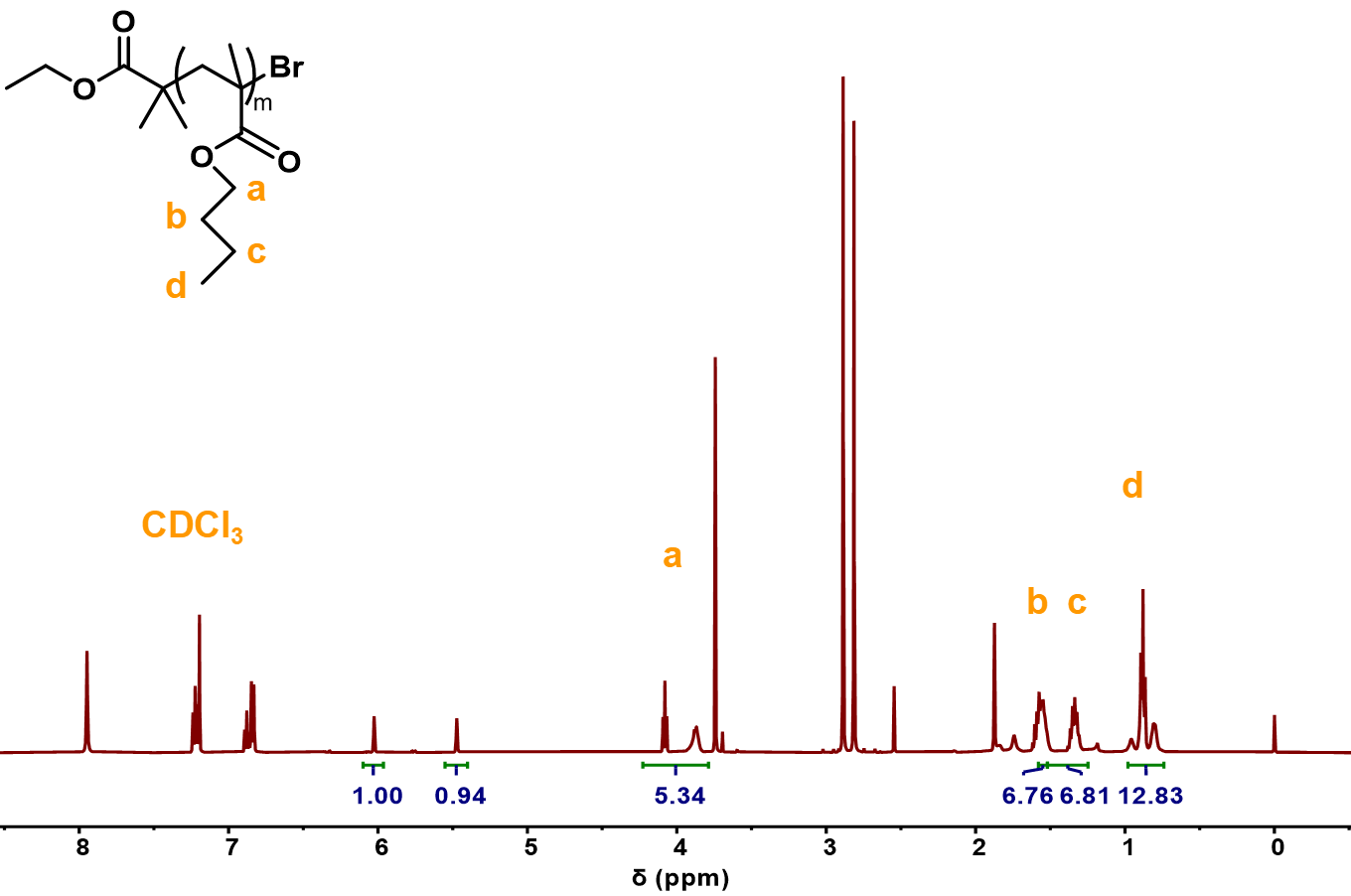


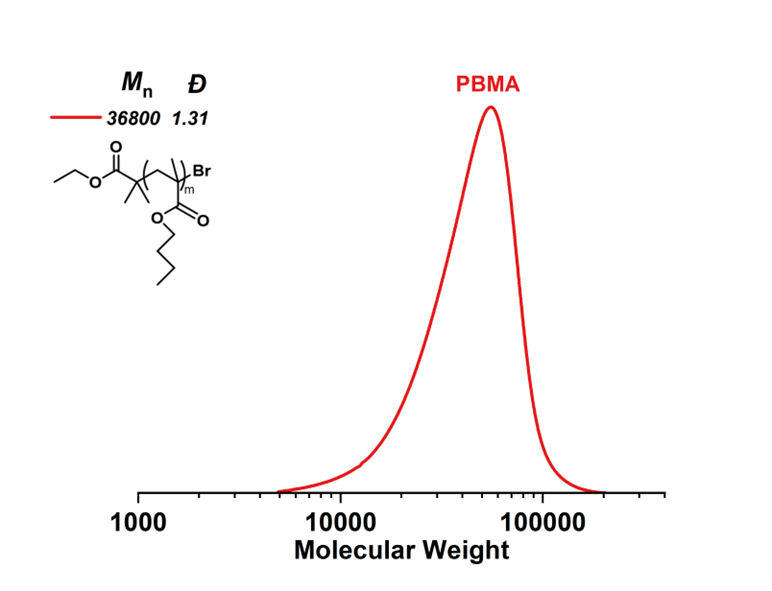


**Figure S25.** Conversion analysis by ^1^H NMR (CDCl_3_) of PBMA of mechanoluminescent hybrid materials and the GPC trace.


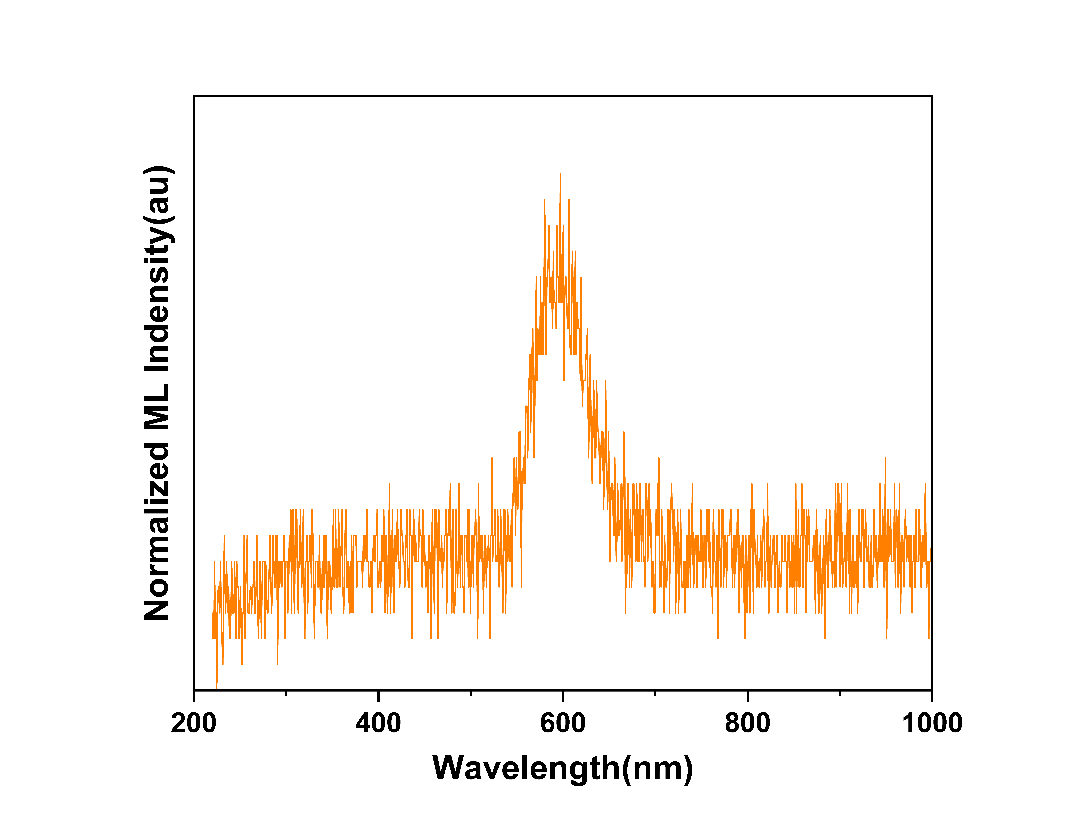


**Figure S26.** ML spectra of PBMA of mechanoluminescent hybrid materials. (1.8 mm thick PBMA)

**
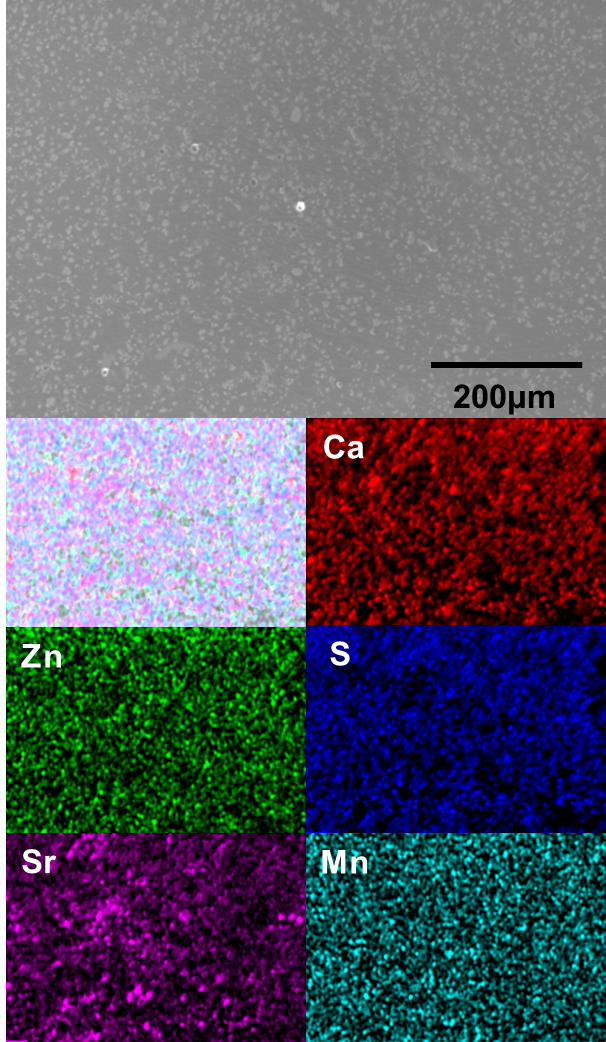
**

**Figure S27.** Scanning Electron Microscope (SEM) image of the mechanoluminescent materials and Elemental mapping of Ca, Zn, S, Sr, and Mn element of inorganic ML powders in mechanoluminescent materials.

**References**

1. Li, X.; Wang, C.; Zheng, Y.; Huang, Z.; Luo, J.; Zhu, M.; Liang, T.; Ren, B.; Zhang, X.; Wang, D.; Ren, Z.; Qu, S.; Zheng, W.; Wei, X.; Peng, D., Smart Semiconductor-Heterojunctions Mechanoluminescence for printable and wearable sports light sources. *Materials & Design* **2023,** *225*, 111589.

2. Xia, J.; Matyjaszewski, K., Controlled/“Living” Radical Polymerization. Atom Transfer Radical Polymerization Catalyzed by Copper(I) and Picolylamine Complexes. *Macromolecules* **1999,** *32*, 2434-2437.
